# *Pseudomonas* effector AvrB is a glycosyltransferase that rhamnosylates plant guardee protein RIN4

**DOI:** 10.1126/sciadv.add5108

**Published:** 2024-02-14

**Authors:** Wei Peng, Nalleli Garcia, Kelly A. Servage, Jennifer J. Kohler, Joseph M. Ready, Diana R. Tomchick, Jessie Fernandez, Kim Orth

**Affiliations:** ^1^ Department of Molecular Biology, University of Texas Southwestern Medical Center, Dallas, TX, USA.; ^2^ Howard Hughes Medical Institute, University of Texas Southwestern Medical Center, Dallas, TX, USA.; ^3^ Department of Microbiology and Cell Science, Institute of Food and Agricultural Sciences, University of Florida, Gainesville, FL, USA.; ^4^ Department of Biochemistry, University of Texas Southwestern Medical Center, Dallas, TX, USA.; ^5^ Department of Biophysics, University of Texas Southwestern Medical Center, Dallas, TX, USA.

## Abstract

The plant pathogen *Pseudomonas syringae* encodes a type III secretion system avirulence effector protein, AvrB, that induces a form of programmed cell death called the hypersensitive response in plants as a defense mechanism against systemic infection. Despite the well-documented catalytic activities observed in other Fido (Fic, Doc, and AvrB) proteins, the enzymatic activity and target substrates of AvrB have remained elusive. Here, we show that AvrB is an unprecedented glycosyltransferase that transfers rhamnose from UDP-rhamnose to a threonine residue of the *Arabidopsis* guardee protein RIN4. We report structures of various enzymatic states of the AvrB-catalyzed rhamnosylation reaction of RIN4, which reveal the structural and mechanistic basis for rhamnosylation by a Fido protein. Collectively, our results uncover an unexpected reaction performed by a prototypical member of the Fido superfamily while providing important insights into the plant hypersensitive response pathway and foreshadowing more diverse chemistry used by Fido proteins and their substrates.

## Teaser

Avirulence protein AvrB is an unprecedented rhamnosyltransferase.

## INTRODUCTION

Bacterial pathogens produce numerous protein effectors/toxins that are delivered into host cells to hijack signaling pathways and assist bacterium growth and infection (*1*, *2*). Host cells have evolved diverse immune response mechanisms to sense and inhibit bacterial infection (*3*–*6*). Plants encode “guardees,” such as RPM1-interacting protein 4 (RIN4), that recognize the presence of specific pathogen effector proteins to trigger the hypersensitive response (HR), a programmed cell death (*3*, *7*–*12*). AvrB, a type III secretion system effector from the plant pathogen *Pseudomonas syringae*, is a member of the Fido (Fic, Doc, and AvrB) family that triggers HR in *Arabidopsis* plants to ward off systemic infection (*7*, *13*–*16*). Upon entry of AvrB into a responsive plant cell, RIN4 is modified by phosphorylation, and this change is sensed by disease resistance proteinresistance to *Pseudomonas syringae* pv. *maculicola* 1 (RPM1) to activate HR resulting in death of the infected cell (*7*, *12*, *17*, *18*). Although AvrB was thought to phosphorylate RIN4, various studies failed to demonstrate that AvrB functions as a kinase-like enzyme (*15*, *19*). Instead, host kinases have been implicated to phosphorylate RIN4 for RPM1 activation (*18*, *20*–*22*). Nevertheless, it remains unknown whether and how AvrB activates host kinases. Therefore, the link between RIN4 sensing AvrB and the plant immune response has been missing.

Fido domain–containing proteins constitute one major superfamily, many of which are bacterial effectors, that uses diphosphate nucleotide charged molecules to mediate diverse posttranslational modifications (*16*, *23*, *24*). *Vibrio* Fido protein VopS provided the first well-studied example of a Fido protein that transfers adenosine monophosphate (AMP) from adenosine triphosphate (ATP) to its substrate (AMPylation) (*25*). This led to the finding of the eukaryotic Fido protein FicD/HYPE that AMPylates its substrate Bip, an endoplasmic reticulum chaperone, that regulates the unfolded protein response (*26*–*28*). Other examples of Fido protein modifications include phosphorylation by Doc using ATP (*29*), phosphocholination by AnkX using cytidine diphosphate choline (*30*, *31*), and UMPylation by AvrAC using UTP (*32*).

The structure (Fido fold) and avirulence activity of AvrB strongly suggest that it may be an enzyme like other Fido proteins but with an unidentified effector activity (*7*, *13*, *15*, *16*, *19*). Our investigations herein reveal the distinct and unexpected activity of AvrB acting as a glycosyltransferase that rhamnosylates residue T166 of *Arabidopsis thaliana* guardee protein RIN4. By elucidating the crystal structures of AvrB and its substrates across different enzymatic states, we provide an explanation for the specificity of substrates and delineate the step-by-step reaction mechanism executed by AvrB in the process of transferring rhamnose to RIN4. Our work uncovers the biochemical function of the enigmatic Fido member AvrB, providing insight into investigations of other AvrB-like proteins with unknown activities.

## RESULTS

### RIN4 is modified by AvrB with a mass shift of +146 Da

**Fig. 1.**
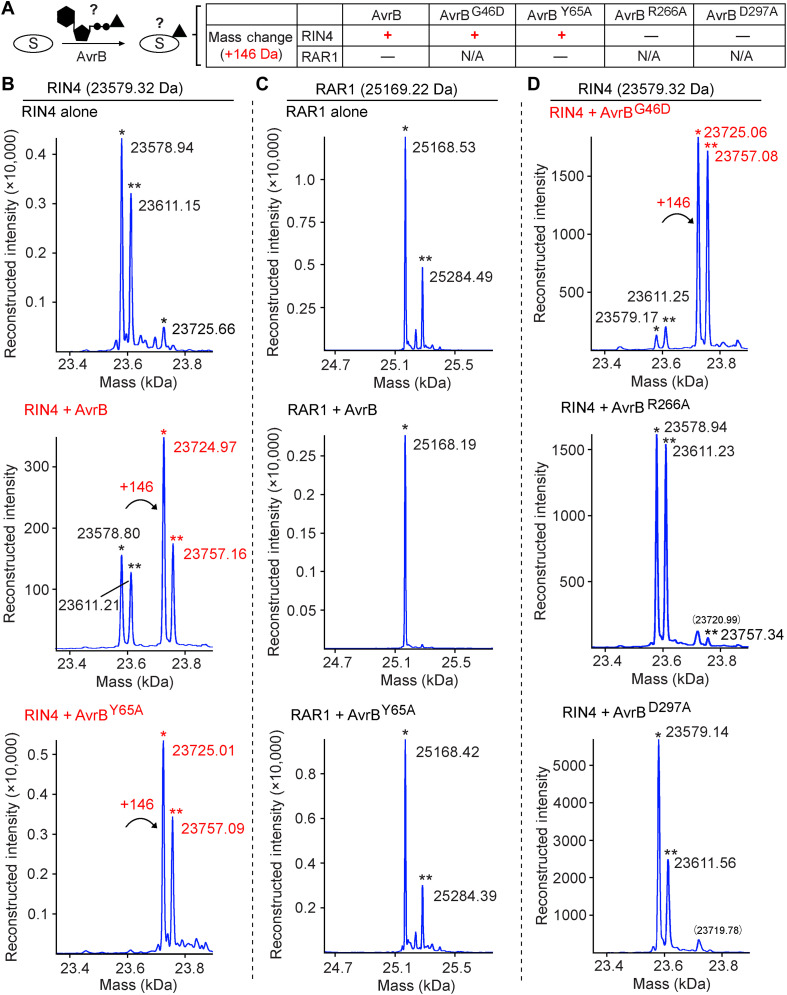
RIN4 coexpressed with AvrB exhibits a mass shift of +146 Da. (**A**) Summary of protein mass shift of RIN4 or RAR1 coexpressed with AvrB in comparison with protein expressed alone. N/A not assessed. Proteins were expressed in BL21 (DE3). (**B**) Intact mass profile of RIN4 expressed alone or with AvrB (WT) or AvrB^Y65A^. “*” symbols in black indicate mass peaks close to the theoretical mass, and “**” symbols in black indicate unknown modification peaks (+32 Da), which happened to be a signature mark for RIN4. “*” or “**” symbols in red indicate mass peaks with a shift of +146 Da. (**C**) Intact mass profile of RAR1 expressed alone or with AvrB or AvrB^Y65A^. “*” symbols indicate mass peaks close to the theoretical mass, and “**” symbols indicate unknown modification peaks. (**D**) Intact mass profile of RIN4 coexpressed with AvrB^G46D^, AvrB^R266A^, or AvrB^D297A^. “*” and “**” symbols indicate similar peaks as in (B).

To test whether AvrB has enzymatic activity and could modify substrates, AvrB and RIN4 or RAR1, another factor involved in host defense and indicated to interact with AvrB (*20*, *33*), were expressed in the *Escherichia coli* strain BL21 (DE3) which may provide unidentified metabolite(s) as cosubstrate(s) for AvrB. The proteins were then purified for intact mass analysis to examine any deviations from their predicted molecular weights (Fig. 1). RIN4 coexpressed with AvrB displayed a clear mass shift of +146 Da compared to RIN4 alone (Fig. 1, A and B). By contrast, the +146-Da shift was not observed with RAR1 or AvrB (Fig. 1, A and C, and fig. S1).

**Fig. 2.**
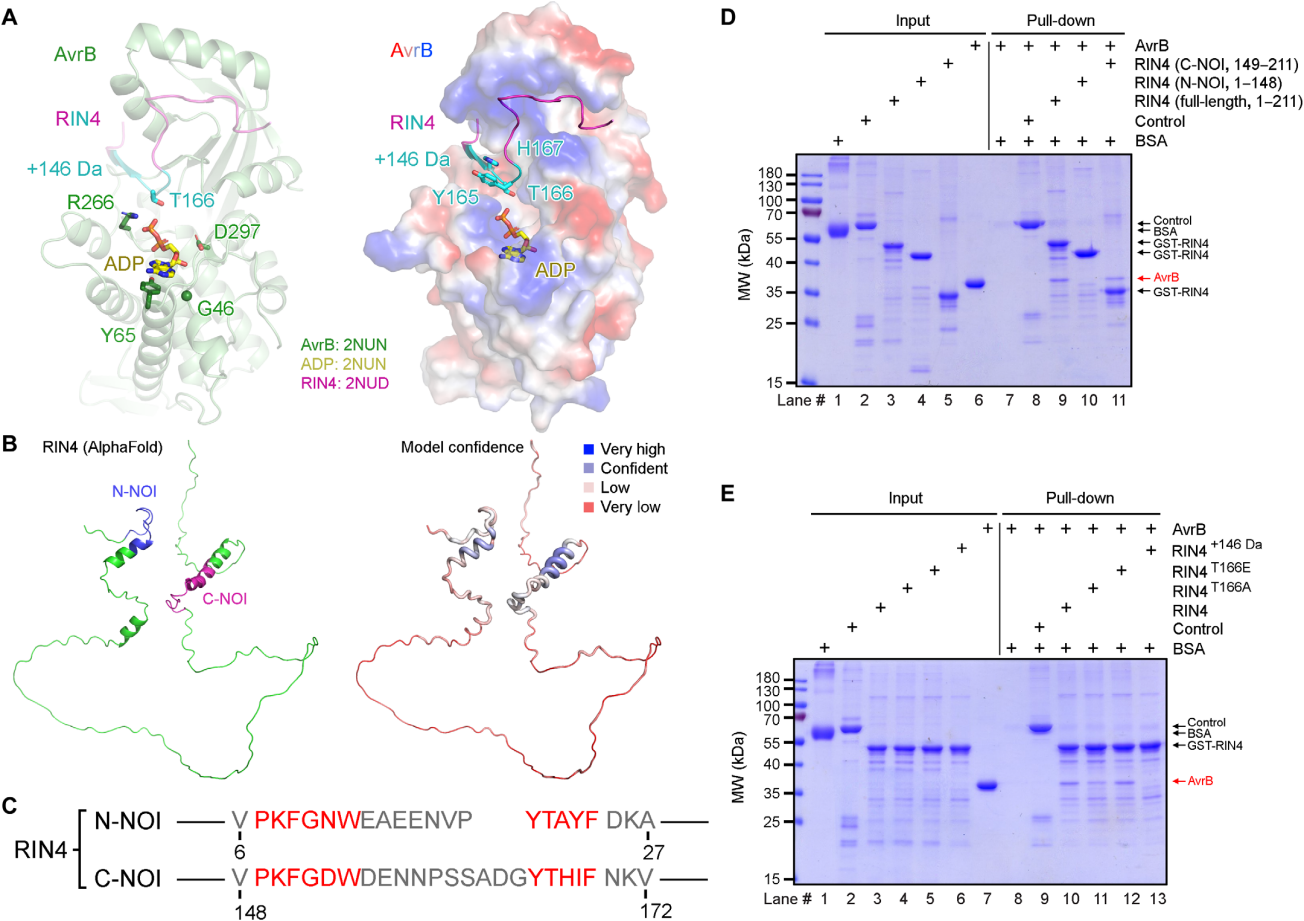
Interaction between RIN4 and AvrB. (**A**) Structure comparison of AvrB bound with ADP [Protein Data Bank (PDB) code: 2NUN] and with RIN4 C-NOI peptide (PDB code: 2NUD). Proteins, residues, and ADP are colored as indicated. Right: Electrostatic surface of AvrB with positive areas in blue, negative in red, and neutral in white (contour level: ±74 *k*_B_T/e); RIN4 residues Y165, T166, and H167 are shown as sticks (side chain); ADP is shown as yellow sticks. (**B**) *A. thaliana* RIN4 structure model predicted by AlphaFold (AF-Q8GYN5-F1). Left: Structure model with the N-terminal and C-NOI domains indicated. Right: Model colored with confidence. (**C**) Amino acid sequence of RIN4 NOI domains with conserved motifs highlighted in red. (**D**) Pull-down assay for testing the interaction between AvrB and RIN4 (full-length, N-NOI, and C-NOI). (**E**) Pull-down assay for testing the interaction between AvrB and RIN4 (WT, T166A, T166E, and +146 Da). MW, molecular weight; BSA, bovine serum albumin.

A few AvrB mutations have been shown to cause loss of AvrB avirulence activity in planta (*19*, *34*). Various AvrB mutants (mapped in Fig. 2A) that may affect cosubstrate binding or enzymatic activity were tested for their ability to induce a mass shift of +146 Da in RIN4 (Fig. 1). AvrB^G46D^ and AvrB^Y65A^ both caused a +146-Da mass increase in RIN4; however, coexpression with mutants of potential catalytic residues, AvrB^R266A^ or AvrB^D297A^, did not induce a mass shift (Fig. 1, A, B, and D). In contrast, RAR1 did not display a mass shift when coexpressed with AvrB^Y65A^ (Fig. 1C), and none of AvrB mutants showed mass shift (fig. S1). These data support the hypothesis that AvrB is an enzyme that specifically modifies the plant guardee protein RIN4 with a mass shift of +146 Da.

### Residue T166 of RIN4 is the site modified by AvrB

RIN4 is a flexible protein with no obvious structural domain, supported by the predicted AlphaFold structure model (Fig. 2B) (*35*). RIN4 has two nitrate-induced (NOI) domains that only contain 20 to 30 amino acids (Fig. 2C) (*12*). Previous studies have shown that AvrB binds to RIN4, and the interaction is mediated by the C-terminal NOI (C-NOI) domain (Fig. 2, A and C) (*19*, *36*). This interaction was confirmed in our hands in a pull-down assay (Fig. 2D). Therefore, the C-NOI could possibly be the region that carried the modification of +146 Da by AvrB. The modified RIN4 with +146 Da lost its ability to interact with AvrB, suggesting the release of RIN4 after modification by AvrB (Fig. 2E).

**Fig. 3.**
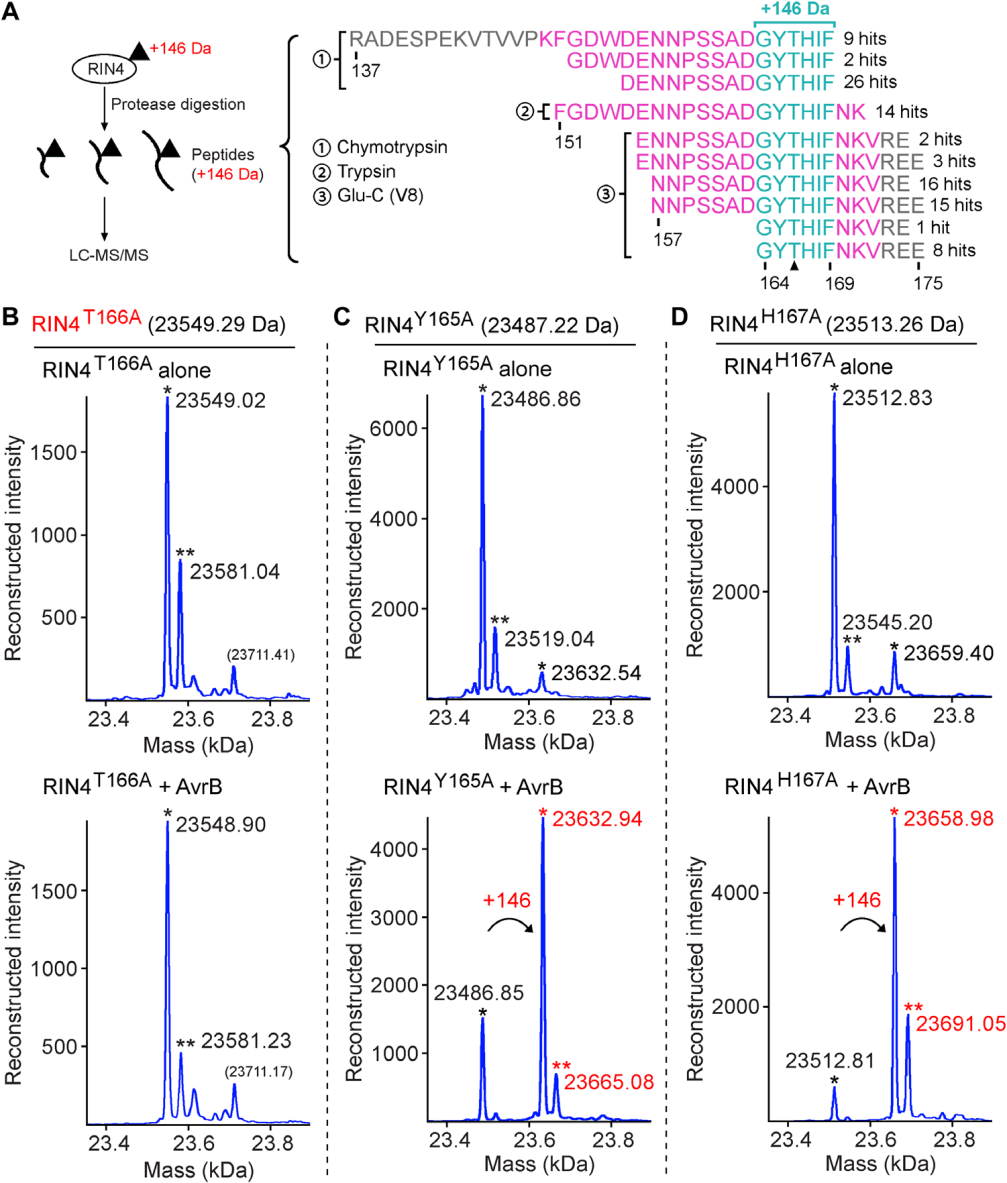
AvrB modifies residue T166 of RIN4. (**A**) LC-MS/MS analysis of RIN4 for identifying modification site of +146 Da. Chymotrypsin, trypsin, and Glu-C (or V8) proteases were used to digest RIN4 and generate various peptides for finding a minimal overlapping region that was modified. RIN4 residues built in the structure model (Fig. 2A) are colored in magenta. Peptides (with a total of 96 MS2 spectral counts or hits) around C-NOI have a minimum overlapping region (in cyan) containing residues G_164_YTHIF_169_. (**B** to **D**) Intact mass profile of mutant RIN4^T166A^ (B), RIN4^Y165A^ (C), or RIN4^H167A^ (D) expressed alone or coexpressed with AvrB. “*” and “**” symbols indicate similar peaks as in Fig. 1B.

Protein liquid chromatography–tandem mass spectrometry (LC-MS/MS) analysis of RIN4 to determine the +146-Da modification site was performed. Because loss of the modification was observed upon fragmentation via Higher Energy Collision Dissociation (HCD), proteases including chymotrypsin, trypsin, and Glu-C (V8) were used to generate various peptides for comparison and finding a minimal overlapping region that contained the modification site. Numerous peptides carrying the modification of +146 Da were identified by LC-MS/MS, with a minimum overlapping region of G_164_YTHIF_169_ (Fig. 3A). Therefore, Y165, T166, and H167 could be the modification site(s). Superimposition of AvrB structures (bound with ADP and with RIN4 C-NOI domain) indicates that a phosphate (or any molecule linked in the cosubstrate) can be transferred from ADP (or other diphosphate nucleotides) to T166 (Fig. 2A) (*19*). Although RIN4^T166A^ could still interact with AvrB (Fig. 2E), it was not modified by AvrB when coexpressed in BL21 (DE3) cells (Fig. 3B). By contrast, the Y165A and H167A RIN4 mutants were still modified by AvrB (Fig. 3, C and D). These data strongly indicate that AvrB modifies RIN4 at residue T166 with a mass increase of +146 Da.

### AvrB is a glycosyltransferase that rhamnosylates RIN4

**Fig. 4.**
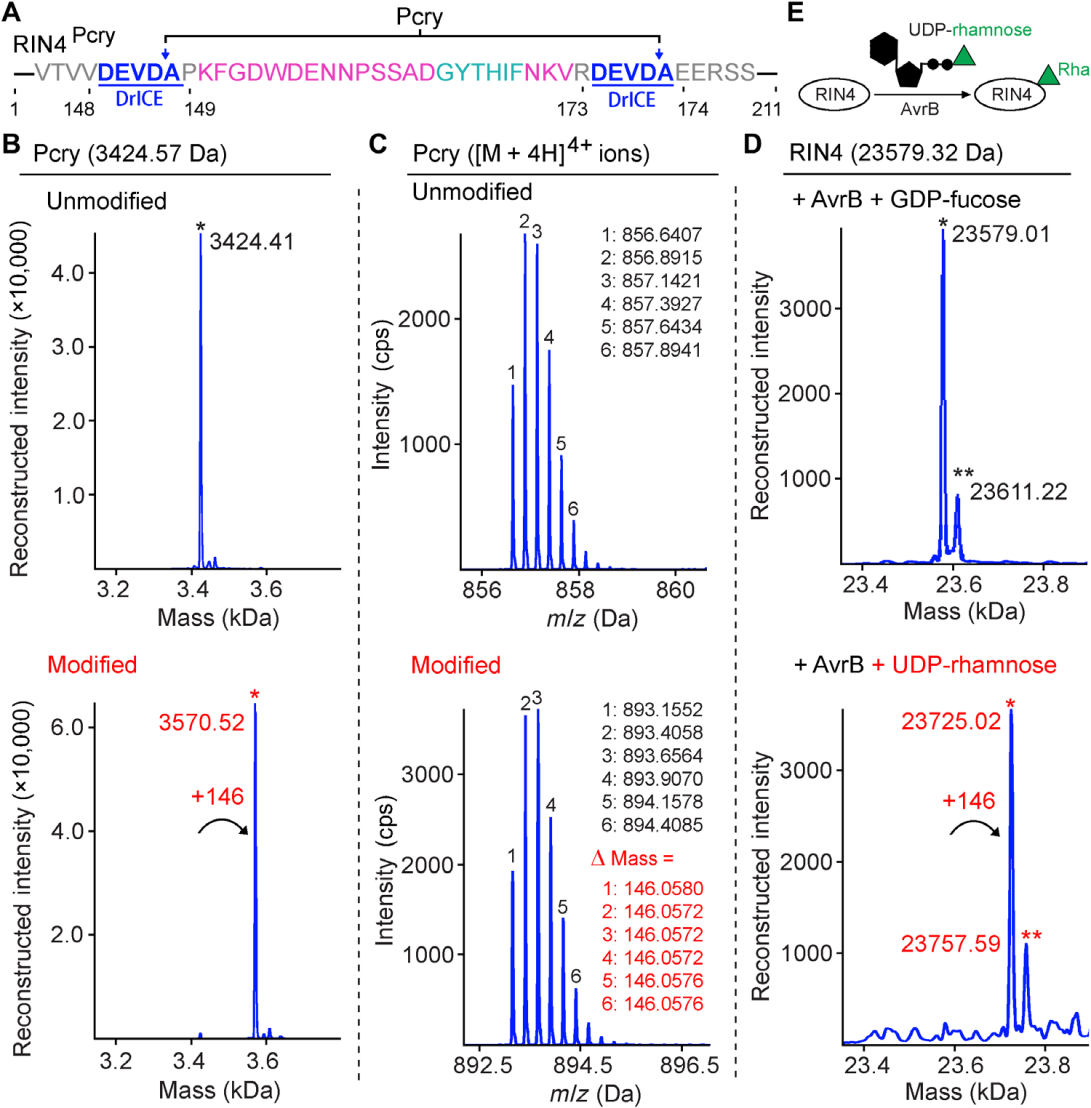
The +146-Da mass increase of RIN4 is caused by rhamnosylation. (**A**) Design of RIN4-Pcry construct. Residues are numbered as in native RIN4 protein. DrICE recognition motifs are colored blue with cutting position indicated by arrows. (**B**) Intact mass profiles of unmodified and modified Pcry peptides (purified after DrICE digestion of GST-RIN4^Pcry^). “*” in black indicates the mass peak close to the theoretical mass. “*” in red indicates the mass peak with a shift of +146 Da. (**C**) Raw data of [M + 4H]^4+^ ions of Pcry peptides in intact mass analysis. Notable isotopic peaks of unmodified and modified peptides are shown. Mass shift (Δ mass in red) was calculated for each isotopic peak pair of unmodified and modified peptides. (**D**) Intact mass profile of RIN4 from in vitro reaction assay. RIN4 was incubated with AvrB and 100 μM cosubstrate (GDP-fucose or UDP-rhamnose). “*” and “**” symbols indicate similar RIN4 peaks as in Fig. 1B. (**E**) Model showing AvrB rhamnosylates RIN4 using UDP-rhamnose as cosubstrate. *m/z*,mass/charge ratio.

For more accurate mass estimation of the +146-Da modification, modified and unmodified RIN4 C-NOI peptides [peptide for crystallization (Pcry)] were purified from reconstructed RIN4 proteins (Fig. 4, A and B; described in Materials and Methods). On the basis of the isotopic distribution of positively charged [M + 4H]^4+^ Pcry peptide ions, the mass shift was estimated to be 146.0572 to 146.0580 Da (Fig. 4C). Calculations with [M + 3H]^3+^ ions, [M + 5H]^5+^ ions, and [M + 5H]^5+^ ions of longer peptides (described in Materials and Methods) resulted in similar mass ranges (fig. S2A). A mass range of 146.0570 to 146.0605 Da covered all the estimations and was used in a search for molecular formulas within this range. Ten hits were returned with elements of C, H, N, O, P, and S (fig. S2B). Among these 10 candidates, two are more likely biologically relevant with C_6_H_10_O_4_ (expected mass of 146.0579 Da) from C_6_H_12_O_5_ (2-deoxy-d-glucose, l-fucose, l-rhamnose, etc.) and C_7_H_6_N_4_ (expected mass of 146.0592 Da) from C_7_H_8_N_4_O (guanine- or adenine-like molecules) (fig. S2C).

All the other four subfamilies of Fido domain–containing proteins use diphosphate nucleotide charged cosubstrates for catalytic reactions, and an ADP molecule could be soaked into the cosubstrate binding pocket of AvrB (*19*, *23*). Therefore, the cosubstrate(s) for AvrB may also contain diphosphate nucleotide(s). To gain insights into potential diphosphate nucleotide(s) used by AvrB, a thermal shift assay was performed in the presence of various nucleotides (fig. S3). Under the condition tested, ADP did not cause an obvious shift in the melting temperature, while UDP caused an obvious shift (~+0.8°C). Likewise, the unmodified Pcry peptide did not result in a thermal shift; however, when it was incubated with UDP we observed a further increase in the melting temperature (~+1.2°C). Mixtures of deoxynucleoside triphosphates (dNTPs) (dATP, dGTP, dTTP, and dCTP) also induced a slight thermal shift, which was not further enhanced by the addition of the Pcry peptide. Thus, UDP appears to be the nucleotide capable of binding to AvrB and may be part of the cosubstrate used by AvrB.

**Fig. 5.**
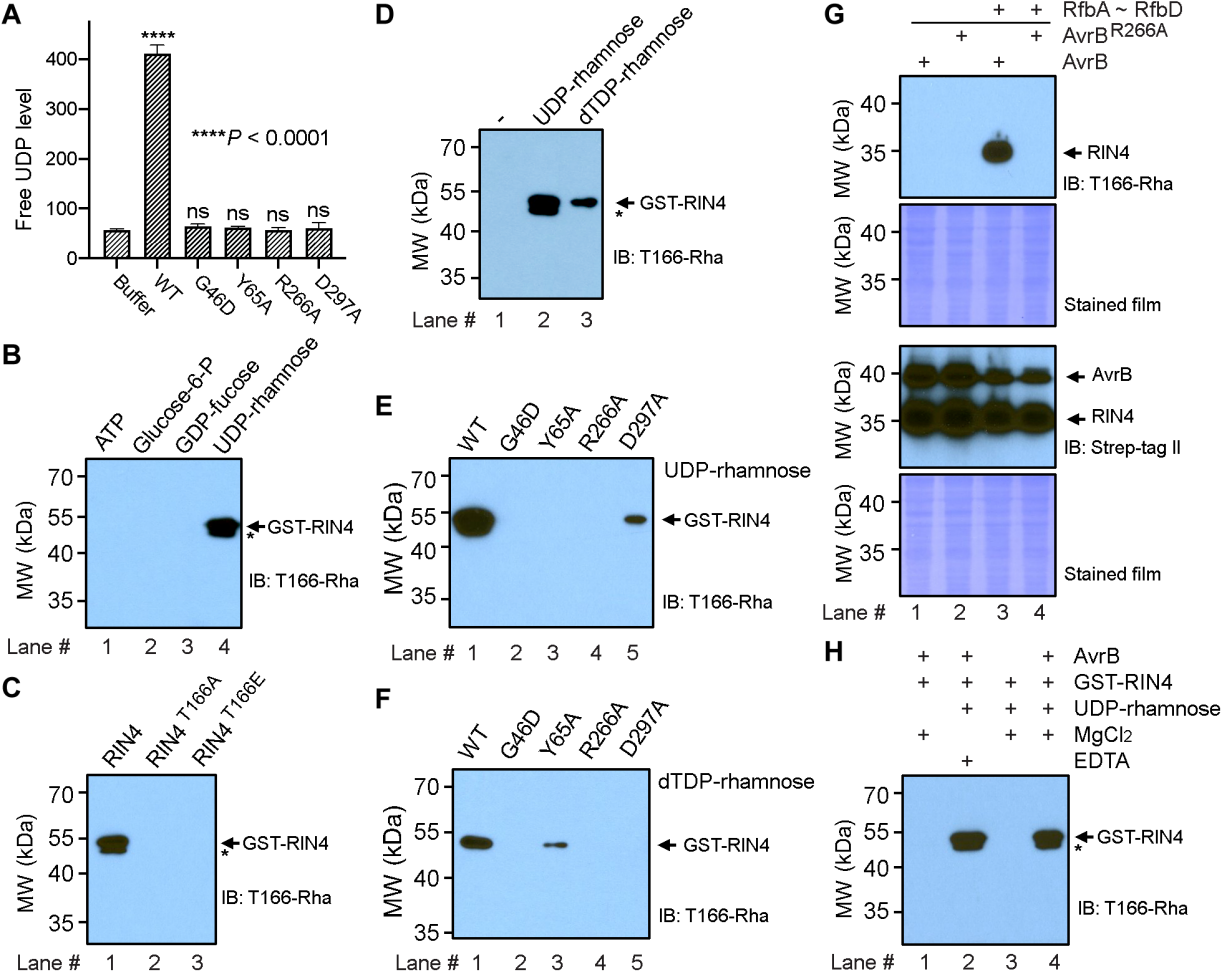
In vitro rhamnosylation of RIN4 catalyzed by AvrB. (**A**) UDP-rhamnose (20 μM) hydrolysis catalyzed by AvrB (~6 nM) in the presence of GST-RIN4 (6 μM) as rhamnose acceptor. Reaction with AvrB (WT or mutant) was compared to buffer control. (**B**) In vitro rhamnosylation of RIN4 by AvrB with cosubstrate (100 μM) of ATP, glucose-6-P, GDP-fucose, or UDP-rhamnose. Rhamnosylated RIN4 was detected by immunoblotting (IB) using T166-Rha–specific antibody. “*” indicates degraded RIN4 in all immunoblotting images. (**C**) Effect of T166A or T166E mutation on rhamnosylation of RIN4 by AvrB (20 μM UDP-rhamnose as cosubstrate). (**D**) RIN4 rhamnosylation with UDP-rhamnose or dTDP-rhamnose as cosubstrate (2 μM). (**E**) Enzymatic activity test for AvrB mutants with UDP-rhamnose (2 μM) as cosubstrate. (**F**) Enzymatic activity test for AvrB mutants with dTDP-rhamnose (2 μM) as cosubstrate. (**G**) Coexpression of RIN4 and AvrB with BL21 (DE3) enzymes (RfbA, RfbB, RfbC, and RfbD) for producing dTDP-rhamnose in HEK 293 T/17 cells. Expression of AvrB and RIN4 was confirmed with anti–Strep-tag II antibody. (**H**) RIN4 rhamnosylation by AvrB in the presence or absence of Mg^2+^ (with 100 μM UDP-rhamnose). ns, not significant.

RIN4 coexpressed with AvrB in human embryonic kidney (HEK) 293 T/17 cells was not modified with a mass increase of +146 Da (fig. S4). This indicates that the cosubstrate for the AvrB modification of RIN4 may not be present in human cells. Because of its source from a plant bacterial pathogen, we hypothesized that AvrB may use UDP-rhamnose, a common plant metabolite (*37*, *38*). We observed a +146-Da mass increase in RIN4 when UDP-rhamnose but not when GDP-fucose. ATP or glucose-6-P was used in in vitro assays (Fig. 4D and fig. S5). Release of UDP was observed after rhamnosylation of RIN4 catalyzed by wild-type (WT) AvrB but not the mutants (Fig. 5A). Thus, AvrB is a glycosyltransferase that uses UDP-rhamnose to rhamnosylate RIN4 on T166 in vitro.

### RIN4^T166-Rha^–specific antibody reveals biochemical properties for AvrB

We developed an antibody specific for rhamnosylated RIN4^T166-Rha^ (described in Materials and Methods). Consistent with our intact mass analysis above, AvrB-mediated rhamnosylation of RIN4 was detected by protein immunoblotting when UDP-rhamnose, but not when ATP, glucose-6-P, or GDP-fucose was used as cosubstrates in the assay (Fig. 5B). As expected, the RIN4 mutants T166A and T166E were unable to be rhamnosylated by AvrB in vitro (Fig. 5C). As discussed above, RIN4 was rhamnosylated by AvrB in *E. coli*. dTDP-rhamnose, instead of UDP-rhamnose, can be synthesized by *E. coli* (*37*, *39*). Hence, dTDP-rhamnose may be an alternative rhamnose donor for AvrB when coexpressed with RIN4 in BL21 (DE3). In vitro RIN4 rhamnosylation was observed when dTDP-rhamnose was used as a cosubstrate, albeit to a lesser extent than UDP-rhamnose (Fig. 5D). The AvrB mutants G46D, Y65A, R266A, and D297A produced decreased anti-RIN4^T166-Rha^ immunoreactivity compared to WT AvrB, with AvrB^D297A^ displaying the highest signal with UDP-rhamnose as the cosubstrate (Fig. 5E) and the Y65A mutant displaying weak activity when dTDP-rhamnose was used (Fig. 5F).

Human cells do not encode enzymes to synthesize or metabolize UDP-rhamnose or dTDP-rhamnose. Therefore, we generated mammalian expression constructs encoding the *E. coli* dTDP-rhamnose biosynthetic enzymes, RfbA, RfbB, RfbC, and RfbD, for expression in HEK 293 T/17 cells. When RfbA, RfbB, RfbC, and RfbD were coexpressed with WT AvrB, but not the R266A mutant, we observed RIN4 rhamnosylation (Fig. 5G). Thus, with the successful reconstitution of the *E. coli* dTDP-rhamnose synthesis pathway, AvrB can rhamnosylate RIN4 in an exogenous system of human cells.

In a rhamnosylation competition/inhibition assay, UDP and, to a lesser extent, a mixture of dNTPs were able to block RIN4 rhamnosylation, whereas the other nucleotides tested displayed little or no effect (fig. S6A). These data are consistent with findings in the thermal shift assay (fig. S3). Similar tests were performed with various sugar or sugar-like molecules, but none of these molecules notably inhibited RIN4 rhamnosylation (fig. S6B). GDP-fucose and UDP-glucose were tested as available sugars linked to diphosphate nucleotides, and neither inhibited RIN4 rhamnosylation (fig. S6C). Therefore, all the observations support that AvrB is a glycosyltransferase that can use UDP-rhamnose or dTDP-rhamnose to modify T166 on RIN4.

### Structural basis for RIN4 rhamnosylation catalyzed by AvrB

**Fig. 6.**
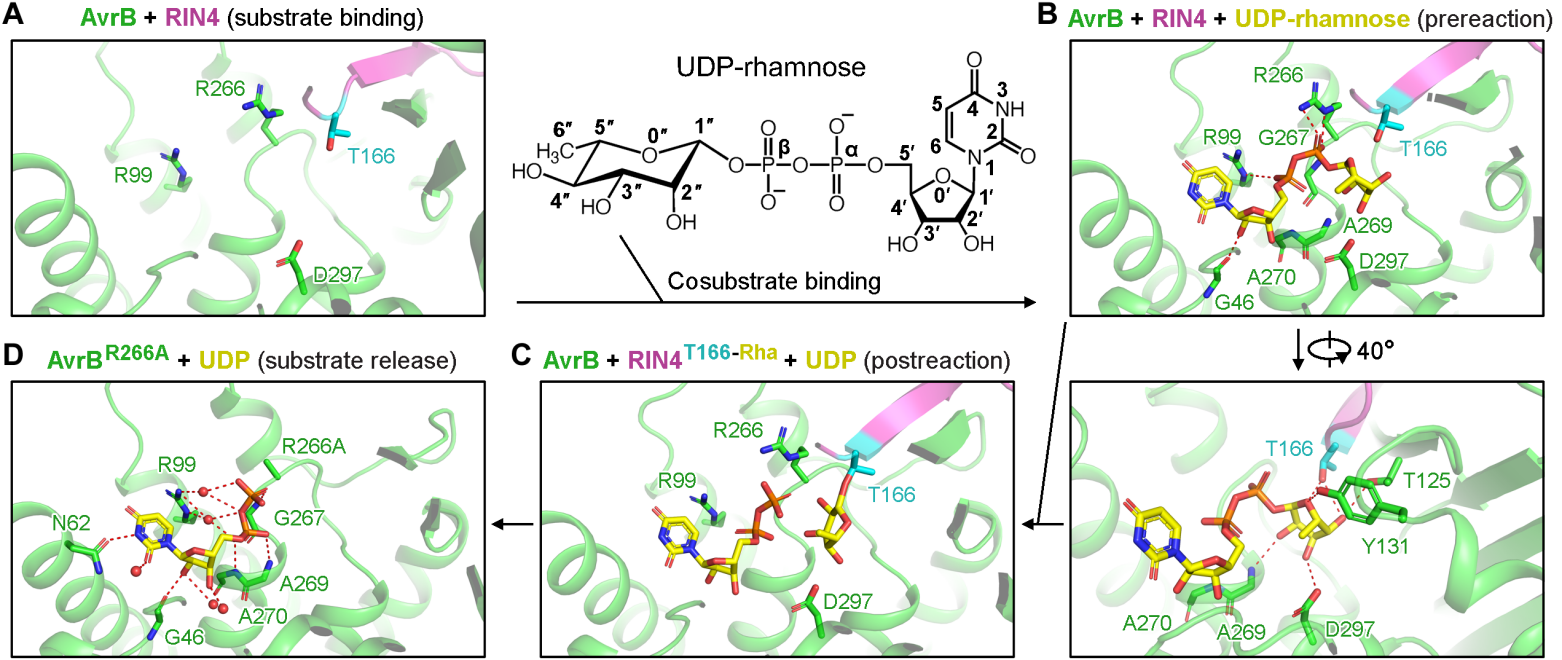
Catalysis mechanisms for RIN4 rhamnosylation by AvrB. (**A**) Crystal structure of AvrB bound with RIN4 (8TXF). UDP-rhamnose atoms are numbered. (**B**) Crystal structure of AvrB bound with RIN4 and UDP-rhamnose (8TWS), representing the prereaction state. Possible hydrogen bonds (or polar contacts) are indicated by red dashed lines. Contacts between UDP and AvrB: O^2^′-G46, α-PO_4_-R99, β-PO_4_-R266, and β-PO_4_-G267. Contacts between rhamnose and AvrB: O^0″^-T166 (RIN4), O^0″^-Y131, O^2″^-A269, O^3″^-D297, O^4″^-T166 (RIN4), and O^4″^-T125. (**C**) Crystal structure of AvrB bound with rhamnosylated RIN4 and UDP (8TWO), representing the postreaction state. (**D**) Crystal structure of AvrB^R266A^ bound with UDP (8TWJ). Contacts between UDP and AvrB: N^3^-N62, O^2^-H_2_O, O^2^′-G46, O^2^′-H_2_O, O^3^′-H_2_O, α-PO_4_-A269, α-PO_4_-A270, α-PO_4_-H_2_O, β-PO_4_-H_2_O, β-PO_4_-G267, and β-PO_4_-R266. AvrB, RIN4, UDP-rhamnose, UDP, and rhamnose are colored as indicated in the figures with water molecules shown as red spheres.

The catalytic motif of AvrB is more divergent when compared to other Fido proteins (*23*), suggesting that AvrB may adopt a different mechanism for catalysis. Unlike the Fido enzyme AnkX that requires Mg^2+^ for catalysis (*31*), AvrB-catalyzed rhamnosylation of RIN4 does not require divalent cations (Fig. 5H). To illustrate the distinct catalytic mechanism of AvrB, we determined crystal structures of AvrB bound with cosubstrates and RIN4 peptides (Fig. 6 and figs. S7 and S8; described in Materials and Methods). Structures of AvrB + RIN4 [similar to a reported structure (*19*)], AvrB + RIN4 + UDP-rhamnose, AvrB + RIN4^T166-Rha^ + UDP, and AvrB^R266A^ + UDP together contribute to deciphering the catalytic mechanisms of AvrB as a rhamnosyltransferase (Fig. 6). Binding of RIN4 places residue T166 in the active site (Fig. 6A). In the cosubstrate binding pocket, UDP-rhamnose adopts a conformation with rhamnose close to the hydroxyl group of T166 (prereaction state, Fig. 6B). Rhamnose is then transferred to T166 (postreaction state, Fig. 6C). In a pull-down assay, RIN4^T166-Rha^ lost the interaction with AvrB, while the T166A and T166E mutants could bind to AvrB (Fig. 2E), indicating release of rhamnosylated RIN4 from AvrB after accepting rhamnose (Fig. 6D).

UDP (free or in UDP-rhamnose) and rhamnose (in UDP-rhamnose) interact with AvrB directly and indirectly through water molecules (Fig. 6, B and D; described in the figure legend). Notably, comparisons of cosubstrates bound with AvrB clearly show that α-PO_4_ in free UDP and ADP (*19*) indirectly interacts with R99 through water molecules (Fig. 6D and fig. S8, A and B). In contrast, α-PO_4_ in UDP of UDP-rhamnose or with RIN4^T166-Rha^ is closer to R99 and forms direct contact with R99, likely due to clash with rhamnose if remaining in the same conformation as in free UDP or ADP (Fig. 6B and fig. S8, C and D) (*19*). The α-PO_4_ likely provides a driving force for priming the reaction by stabilizing the sugar moiety. dTDP-rhamnose could also be exploited by AvrB to rhamnosylate RIN4 as discussed above. ADP could be soaked into the cosubstrate binding pocket of AvrB (*19*). These findings together suggest that although UDP-rhamnose may be the most favorable cosubstrate, other natural or artificial compounds with minor differences (thus interacting with AvrB similarly with UDP-rhamnose) may also be accommodated and used by AvrB.

## DISCUSSION

The identified biochemical activity of the avirulence protein AvrB as a rhamnosylator adds to the catalytic versatility of the Fido domain–containing protein superfamily. AvrB represents a previously unidentified class of protein glycosyltransferase that modifies serine/threonine residues (O-linked rhamnosylation), distinct from the only known protein rhamnosyltransferase EarP (containing two Rossmann folds and belonging to glycosyltransferase superfamily B) that modifies an arginine residue (N-linked rhamnosylation) (*40*–*44*). As a prototypical enzyme, AvrB’s biochemical activity may provide insights into investigations of similar proteins. In support of this, we found another effector protein AvrC (*45*) undergoes automodification with a mass shift of +146 Da (fig. S9), indicating a likely rhamnosylation event.

We observe that the plant pathogen *P. syringae* expresses an effector that uses a host specific metabolite. Various species metabolites, including *A. thaliana*, were prepared as cosubstrate sources in a rhamnosylation assay (fig. S10). As expected, metabolites from *Arabidopsis* leaf and *E. coli* strain BL21 (DE3) were able to facilitate RIN4 rhamnosylation by AvrB. However, the DH5α bacterial sample did not result in obvious RIN4 rhamnosylation, consistent with the finding that DH5α has a deficient *rfbD* gene that encodes the enzyme required for the last dTDP-rhamnose synthesis step (*46*). The *E. coli* strain Mach1 likely has a gene deficiency as well, while TOP10 and Rosetta (DE3) contain functional genes for producing dTDP-rhamnose, enabling AvrB-mediated rhamnosylation. *P. syringae* pv. *tomato* DC3000D28E (*47*), HEK 293 T/17, yeast, and insect cell (Sf21 or High Five) samples were not able to provide a cosubstrate for in vitro rhamnosylation. Metabolites from the AvrB host, *P. syringae*, were unable to mediate rhamnosylation, thus providing a predicted spatiotemporal control of AvrB activity (*1*). The results imply that the bacterial effector AvrB rhamnosylates RIN4 with nucleotide sugar donor (UDP-rhamnose) available in planta.

Injection of AvrB into a responsive plant cell causes RPM1-mediated HR, which is correlated with RIN4 phosphorylation (*7*, *17*, *18*). However, direct phosphorylation of RIN4 by AvrB has not been detected in the current and previous studies (*15*, *19*). Instead, studies have suggested that host kinases MPK4 and RIPK (RPM1-induced protein kinase) or other RLCKs (receptor-like cytoplasmic kinase) are responsible for RIN4 phosphorylation (*18*, *20*–*22*). RIN4 phosphorylation mutant mimics (T166D and T166E) can induce HR, supporting that RIN4 phosphorylation may be a trigger signal for HR (*17*, *18*). However, other RIN4 modifications performed by effectors and other mutations of RIN4 T166 are known to trigger HR. For example, AvrRpm1, an ADP ribosyltransferase that modifies RIN4 (N11 and D153), induces T166 phosphorylation and triggers RPM1-dependent immune response (*7*, *10*, *18*). AvrRpm1-triggered HR is not dependent on T166 phosphorylation (*11*, *17*). In addition, bacterial acetyltransferase effectors (HopZ5 and AvrBsT) which directly modify T166 of RIN4 with an acetyl moiety also trigger RPM1-dependent defense (*11*). An acetylation mimic mutant of RIN4, T166I, can also trigger RPM1-dependent immunity (*11*). Therefore, many modifications, including but not dependent on T166 phosphorylation, can induce RPM1 activation. Here, we answer the long-standing question about the biochemical activity for the avirulence protein AvrB, revealing previously unknown chemistry for the Fido superfamily of enzymes and shedding light on the AvrB-RIN4-RPM1 axis as one of the most extensively studied plant immune response pathways.

## MATERIALS AND METHODS

### Experimental design

The objective of the study is to reveal the biochemical activity of AvrB as a potential bacterial effector enzyme. Mass spectrometry, biochemical assays, and protein crystallography are used to identify the enzymatic activity and elucidate the catalysis mechanisms.

### Protein expression and purification

The cDNA encoding *P. syringae* AvrB (WT or mutant) was cloned into the pET-29b vector with a C-terminal 6xHis tag. The cDNA encoding *A. thaliana* RIN4 (WT or mutant) was cloned into a modified pET-15b vector with an N-terminal 6xHis tag followed by a DrICE protease cutting site (DEVD^A). The plasmid was transformed into *E. coli* strain BL21 (DE3) for protein expression. For coexpression, AvrB and RIN4 plasmids were cotransformed into BL21 (DE3). The bacteria were cultured at 37°C. When optical density at 600 nm reached ~1.0, the temperature was adjusted to 22°C, and 0.2 mM isopropyl-β-d-thiogalactopyranoside was added for overnight induction. Cells were collected by centrifugation, resuspended in lysis buffer B1 [25 mM tris-HCl (pH 8.0) and 150 mM NaCl]. After cell disruption and removal of cell debris by centrifugation at 22,000*g* for 1 hour, the supernatant was loaded to Ni^2+^-NTA resin (Qiagen). The resin was washed by buffer B2 (lysis buffer B1 with 300 mM NaCl) and sequentially by buffer B3 (lysis buffer B1 containing 10 mM imidazole). Protein bound to the resin was eluted by buffer B4 (lysis buffer B1 containing 250 mM imidazole). 6xHis tag of RIN4 was removed by DrICE. Intact mass analysis was carried out to examine the protein mass as below. The eluted protein was dialyzed against buffer B5 [25 mM Hepes (pH 7.4) and 150 mM NaCl] with 7- or 10-kDa Slide-A-Lyzer Dialysis Cassettes (Thermo Fisher Scientific). The protein was flash-frozen by liquid nitrogen and stored at −80°C for later use.

AvrB for crystallization was obtained similarly with AvrB cDNA subcloned into a modified pET-15b vector as above. 6xHis tag was removed by DrICE during dialysis against buffer B6 (lysis buffer B1 with 50 mM NaCl). Glutathione *S*-transferase (GST)–RIN4 protein was obtained similarly with RIN4 cDNA cloned into a modified pGEX-4 T-2 vector with a C-terminal 6xHis tag. The protein was purified using Ni^2+^-NTA resin as above. Ion exchange with a MonoQ column was performed to separate GST-RIN4^T166-Rha^ from AvrB coexpressed.

### RIN4 peptide purification

For purification of RIN4 C-NOI Pcry (shown in Fig. 4A) or for antibody production (Pab), RIN4^Pcry^ or RIN4^Pab^ was cloned into a modified pGEX-4 T-2 vector. DrICE cutting sites were introduced before residue 149 and after residue 173 for Pcry peptide and before residue 149 and after residue 178 for Pab peptide (all residues after S178 were removed in RIN4^Pab^). GST-RIN4^Pcry^ or GST-RIN4^Pab^ plasmid was transformed alone or together with the AvrB^Y65A^ plasmid into BL21 (DE3). After induction for protein expression, the lysate supernatant was loaded to glutathione agarose resin (Thermo Fisher Scientific). After washing with buffer B6 for peptide Pcry or phosphate-buffered saline for peptide Pab, DrICE protease was added for on-column digestion. Solution containing released peptide was then loaded to Ni^2+^-NTA resin for removal of His-tagged fragment and DrICE protease. Purified peptide was collected for later use.

### Negative purification of RIN4^T166-Rha^–specific antibodies

Antibodies were raised in two rabbits using rhamnosylated Pab peptide (as described above) with a 70-day protocol (Thermo Fisher Scientific). GST-RIN4^Pab^ plasmid was transformed alone into BL21 (DE3) without AvrB plasmid for protein expression. The lysate supernatant was loaded into glutathione agarose resin. Rabbit serum sample containing RIN4 antibodies was loaded to the resin for removal of antibodies, which bind to unmodified RIN4, while the effluent containing RIN4^T166-Rha^–specific antibodies was collected for later use. AB4724 after negative purification was more specific and had no or little background against non-rhamnosylated RIN4 protein compared to AB4725. Negatively purified AB4724 was used to detect rhamnosylated RIN4.

### Pull-down assay

Ten micrograms of bait protein (with GST tag) and 10 μg of prey protein were mixed with 10 μl of glutathione agarose resin in 500 μl of pull-down buffer (containing 5 mg of bovine serum albumin for blocking). Pull-down buffer was composed of 25 mM Hepes (pH7.4), 150 mM NaCl, 1 mM dithiothreitol (DTT), and 0.1% Triton X-100. Samples were incubated at 4°C for 30 min. After centrifugation at 1000*g* for 1 min, the supernatant was discarded. The resin was then washed three times with 500 μl of pull-down buffer. SDS sample buffer was mixed with the remaining resin for SDS–polyacrylamide gel electrophoresis and Coomassie Brilliant Blue staining.

### AvrB and RIN4 coexpression in HEK 293 T/17 cells

cDNA of AvrB or RIN4 was cloned into a modified pcDNA 3.1D/V5-His-TOPO vector with an N-terminal Twin-Strep-tag. cDNA of *rfbA*, *rfbB*, *rfbC*, or *rfbD* was cloned into the vector with a C-terminal Flag tag. HEK 293 T/17 cells were cotransfected with plasmids for 2 days using PolyJet reagent (SignaGen Laboratories) following the manufacturer’s manual.

### Mass spectrometry

Protein intact mass analysis was performed following a previous protocol (*48*). Reducing reagent DTT was added into protein samples at a final concentration of 10 mM before intact mass analysis. Protein LC-MS/MS analysis of RIN4 to determine the +146-Da modification site was performed similarly as reported (*48*). Because loss of the modification was observed upon fragmentation via HCD, proteases including chymotrypsin, trypsin, and Glu-C (V8) were used to generate various peptides for comparison and finding a minimal overlapping region that contained the modification site.

### Thermal shift assay

The thermal shift assay was performed similarly as previously reported with modifications (*49*). Triplicate 25-μl reaction systems contained AvrB protein (0.2 mg/ml; ~5 μM). Reaction buffer contained 25 mM Hepes (pH 7.4), 100 mM NaCl, 1 mM compound, RIN4 peptide Pcry (±0.06 mg/ml; ~18 μM), and 1:500 diluted SYPRO Orange Protein Gel Stain (Sigma-Aldrich). Reactions were performed in a 96-well polymerase chain reaction plate with a Bio-Rad CFX96 Real-Time System. Samples were subjected to a gradient of temperature 10° to 90°C (hold 5 s, increase 0.5°C, rate 0.5°C/s). Fluorescent signals were recorded using a fluorescence resonance energy transfer channel. Melting curves were normalized for each replicate and plotted in GraphPad Prism software. The temperature at the derivative peak was set as the melting temperature, calculated by Bio-Rad CFX Maestro Software.

### In vitro rhamnosylation assay

Rhamnosylation reactions were carried out in a buffer solution containing 25 mM Hepes (pH 7.4), 100 mM NaCl, and 5 mM DTT. Standard reactions included 0.4 μg of AvrB and 0.8 μg of GST-RIN4 in a final volume of 25 μl with 20 μM UDP-rhamnose (MedChemExpress). Cosubstrate concentration varied in each individual set of reactions as indicated in figure legends and below. Reactions were performed at room temperature for 30 min and stopped by the addition of 25 μl of 2× SDS sample buffer. SDS-PAGE and Western blot were conducted to detect rhamnosylated RIN4 with RIN4^T166-Rha^–specific antibody.

Rhamnosylation inhibition reactions were carried out with 0.2 μM UDP-rhamnose and 1 mM nucleotide or sugar. AvrB (0.01 μg) and 0.4 μg of GST-RIN4 were included in the reactions.

Rhamnosylation reactions with cell/tissue metabolites were performed similarly as above. Cell/tissue lysate was heated (>95°C, 10 min), and protein precipitation was removed by centrifugation. The supernatant was normalized (to ~2.5 mg/ml of protein before heating), and 10 μl was used in each reaction as cosubstrate source.

### UDP-rhamnose hydrolysis assay

UDP-rhamnose hydrolysis assay was carried out using the UDP-Glo Glycosyltransferase Assay kit (Promega) following the manual. Briefly, 0.4 μg (~0.5 μM) of AvrB and 8 μg of GST-RIN4 (~6 μM) were included in the reaction in the same buffer as in rhamnosylation assay above (final volume of 25 μl). UDP-rhamnose (20 μM) was included as donor. Reactions were performed at room temperature for 30 min. Equal volume of UDP detection reagent was then added and incubated for additional 60 min. A BMG Labtech CLARIOstar Plus Microplate Reader was used to record the luminescence signals. The mean values of triplicate readings for various groups of samples were analyzed and compared by ordinary one-way analysis of variance (ANOVA) and Tukey test (*P* < 0.05) using GraphPad Prism software.

### Plant leaf sample preparation

Plant leaves were harvested, frozen in liquid nitrogen, and ground with liquid nitrogen. A total of 1.5 times (milliliters per gram) of cold extraction buffer [50 mM Hepes (pH 7.4), 150 mM NaCl, protease inhibitor cocktail, 1 mM phenylmethylsulfonyl fluoride, and 5 mM DTT] was added. After incubation on ice for 30 min, insoluble materials were removed by centrifuge at 4°C for 10 min at >20,000*g*. The supernatant was collected for preparing metabolites after heating (>95°C, 10 min) and removal of denatured proteins.

### Western blot

Samples were applied to SDS-PAGE and transferred to polyvinylidene difluoride membrane or nitrocellulose membrane for immunoblotting. RIN4^T166-Rha^–specific antibody was used to detect modified RIN4 in combination with secondary anti-rabbit horseradish peroxidase antibody.

### Crystallization and x-ray data collection

Crystals of AvrB alone and bound with RIN4 peptide were obtained following previously reported protocols with modifications (*15*, *19*). Recombinant AvrB protein and rhamnosylated RIN4 peptide (Pcry) were purified as above. AvrB was concentrated to ~10 mg/ml with or without rhamnosylated RIN4 peptide (molar ratio of 1:10) in the presence of 5 mM DTT. Protein crystallization was performed with a hanging drop vapor diffusion method. Crystals appeared 7 days after setting up crystallization trays.

Representative crystal structures and the corresponding crystallization conditions are listed in fig. S7. Apo AvrB^R266A^ crystals were obtained at 4°C [100 mM glycine (pH 8.9 to 9.5) and 27 to 34% PEG 550 MME (polyethylene glycol monomethyl ether 550)]. Soaking of AvrB^R266A^ crystals (4°C, overnight) was performed with 5 mM UDP-rhamnose or dTDP-rhamnose in cryoprotectant buffer [100 mM tris (pH 7.7), 50 mM NaCl, 32 to 36% PEG 550 MME, and 10% ethylene glycol]. Soaked AvrB^R266A^ crystals were harvested and frozen in liquid nitrogen. UDP-rhamnose was likely hydrolyzed after soaking (fig. S7D), and dTDP-rhamnose did not bind in the cosubstrate pocket of AvrB (fig. S7E).

AvrB + RIN4 crystals were obtained at 20°C [100 mM tris (pH 7.5 to 7.8) and 27 to 32% PEG 550 MME]. Native AvrB + RIN4 crystals were harvested and frozen in liquid nitrogen after transferred into cryoprotectant buffer. A structure derived from a native AvrB + RIN4 crystal indicated T166 of RIN4 was not rhamnosylated (fig. S7A).

Soaking of AvrB + RIN4 crystals (20°C, 4 hours or overnight) was performed with 5 mM UDP-rhamnose in cryoprotectant buffer as above. Soaked AvrB + RIN4 crystals were harvested and frozen in liquid nitrogen. Soaked AvrB + RIN4 crystals were found be highly anisotropic.

Crystal diffraction data were collected at the Advanced Photon Source (APS) beamline 19-ID and the Advanced Light Source (ALS) beamline 2.0.1. Data were indexed, integrated, and scaled using the HKL-3000 program package (*50*) for data collected at APS and processed with Xia2 (*51*) and DIALS (*52*) for data collected at ALS. Data collection statistics are provided in table S1.

### Structure refinement

The structures of AvrB bound with ADP [Protein Data Bank (PDB) accession code: 2NUN] and AvrB bound with RIN4 peptide (PDB accession code: 2NUD) were used as model for molecular replacement in the program Phenix (*53*). Models were manually adjusted in the program COOT (*54*). Structure refinement was performed in the program PHENIX (*53*). The statistics of the geometries of the models were generated using MolProbity (*55*). Structure figures were prepared with PyMOL (The PyMOL Molecular Graphics System, version 2.4, Schrödinger LLC).

## Supplementary Materials

### This PDF file includes:

Figures S1 to S10 Table S1 References
