# Supplementary material for: Pseudomonas effector AvrB is a glycosyltransferase that rhamnosylates plant guardee protein RIN4
Source: Sci Adv. 2024 Feb 14;10(7):eadd5108. doi: 10.1126/sciadv.add5108 (PMC10866546; doi:10.1126/sciadv.add5108)
Supplement: Supplementary file 1 — Figures S1 to S10 Table S1 References [file sciadv.add5108_sm.pdf]

Science Advances
AAAS

Supplementary Materials for

Pseudomonas effector AvrB is a glycosyltransferase that rhamnosylates plant guardee protein RIN4

Wei Peng *et al.*

Corresponding author: Kim Orth, kim.orth@utsouthwestern.edu

Sci. Adv. **10**, eadd5108 (2024)
DOI: 10.1126/sciadv.add5108

This PDF file includes:

Figs. S1 to S10
Table S1
References

Supplemental Figures and Table

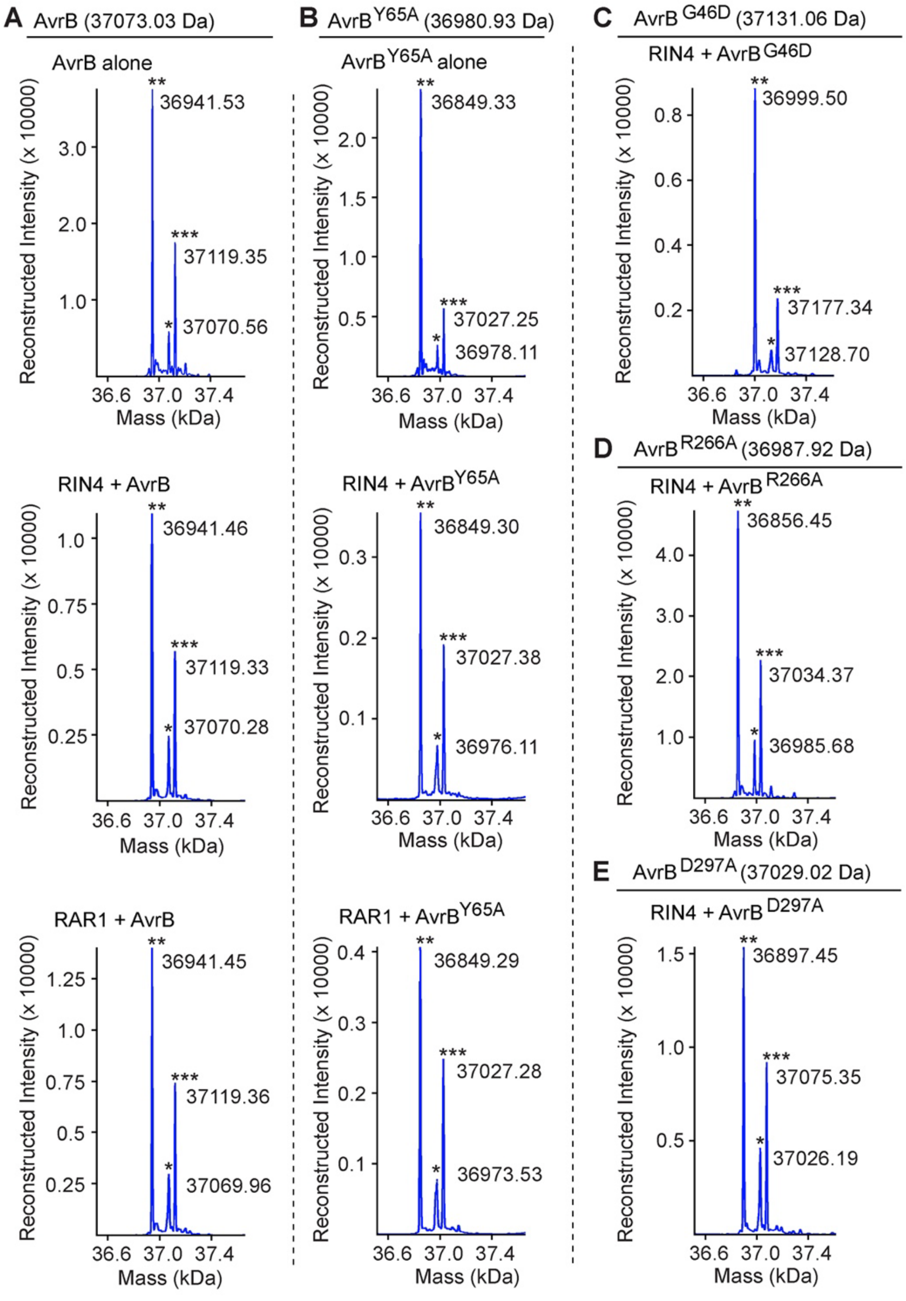

Fig. S1. Intact mass analysis of AvrB. (A) Intact mass profile of AvrB (WT) expressed alone or with RIN4 and RAR1. "*" symbols indicate mass peaks close to the theoretical mass; "**" symbols indicate peaks likely caused by removal of initiator methionine; "***" symbols indicate peaks likely caused by gluconoylation (compared to "**"). (B) Intact mass profile of AvrB^{Y65A} expressed alone or with RIN4 and RAR1. "*", "**", and "***" symbols indicate similar peaks as in (A). (C, D, E) Intact mass profiles of AvrB^{G46D}, AvrB^{R266A}, and AvrB^{D297A} co-expressed with RIN4. "*", "**", and "***" symbols indicate similar peaks as in (A).

A

Pcry:
Δ mass ($[M+3H]^{3+}$ ions):
146.0571 ~ 146.0583 Da
Δ mass ($[M+5H]^{5+}$ ions):
146.0575 ~ 146.0595 Da

Pab:
Δ mass ($[M+5H]^{5+}$ ions):
146.0570 ~ 146.0605 Da

B

Molecular Formula	Monoisotopic mass (146.0520 - 146.0655 Da)
H_4N_9O	146.0539
$C_2H_6N_6O_2$	146.0552
$C_4H_8N_3O_3$	146.0566
$C_6H_{10}O_4$	146.0579
$C_7H_6N_4$	146.0592
$C_3H_9N_5P$	146.0596
C_9H_8NO	146.0606
$C_5H_{11}N_2OP$	146.0609
$C_4H_{10}N_4S$	146.0626
$C_6H_{12}NOS$	146.0640

C

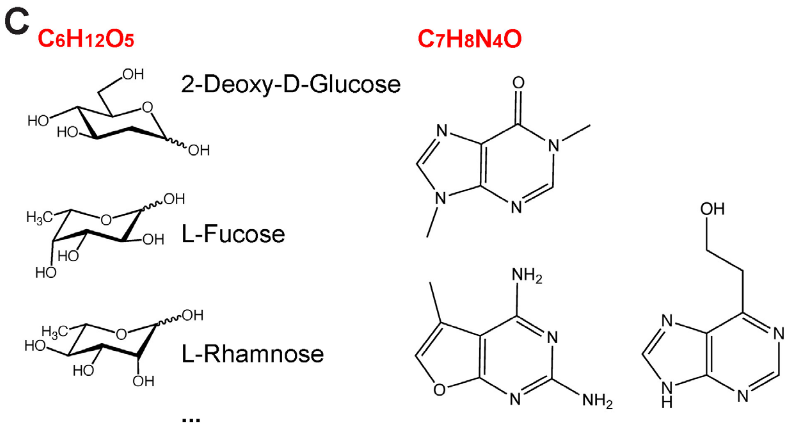

Figure S2. Possible modifications with +146 Da mass shift. (A) Summary of peptide mass shifts for $[M+3H]^{3+}$ and $[M+5H]^{5+}$ ions of unmodified and modified Pcry peptides, and for $[M+5H]^{5+}$ ions of unmodified and modified Pab peptides (described in Methods). Mass shift was calculated similarly as in Fig. 4C. (B) Summary of molecular formulas in the mass range of 146.0570~146.0605 Da. Search was performed with elements of C, H, N, O, P, and S at https://www.chemcalc.org/mf-finder (*56*). (C) Possible molecular structures that are likely biologically relevant and can result in modification with a mass shift of +146 Da (after loss of H_2O). Searches were performed at http://www.chemspider.com/, https://coconut.naturalproducts.net/, and https://ecmdb.ca/.

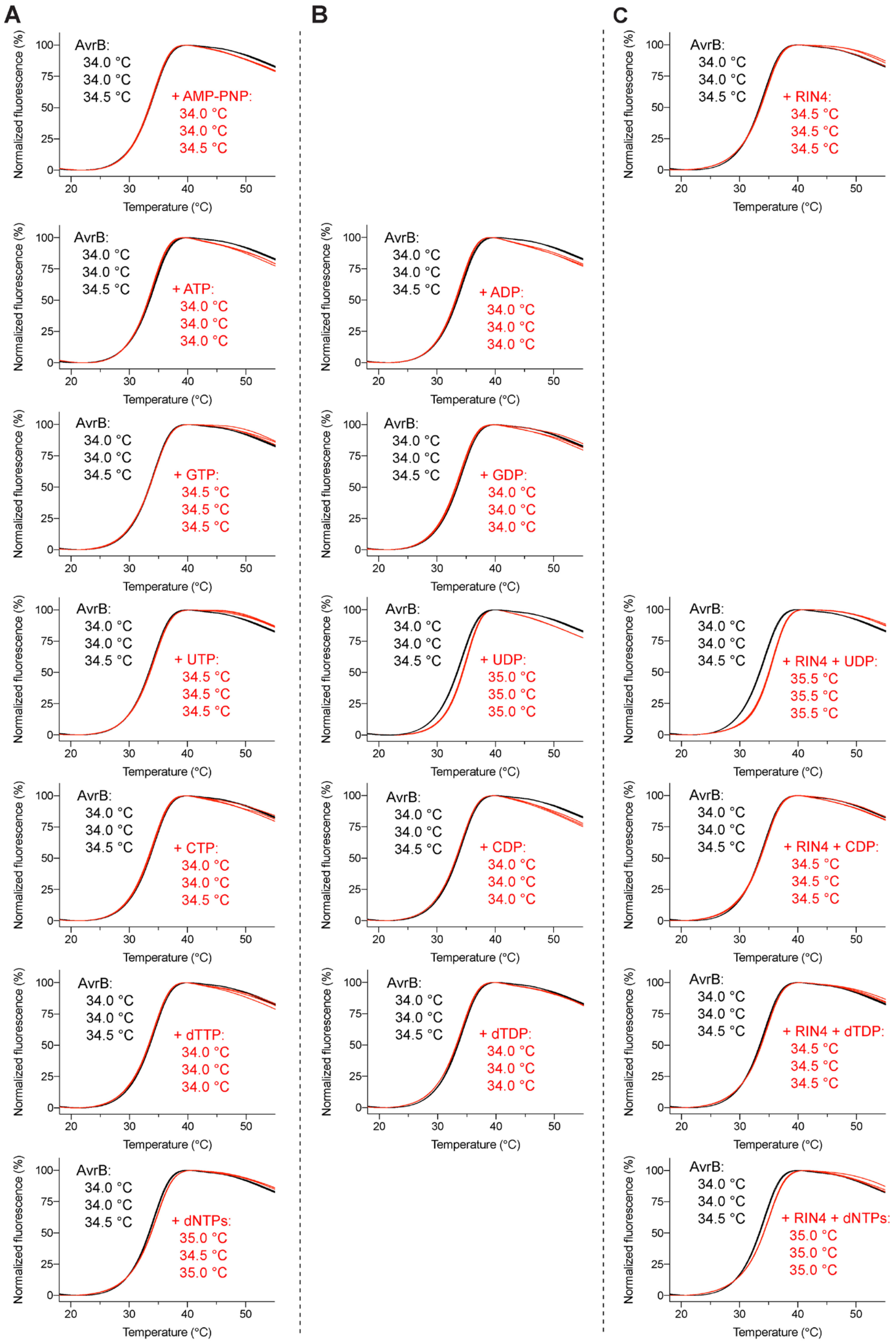

Figure S3. Thermal shift assay for testing interaction between AvrB and nucleotides. (A) Curves for AvrB (5 μM) alone (control) and with 1 mM nucleotide of AMP-PNP, ATP, GTP, UTP, CTP, dTTP, or dNTPs (1 mM each). (B) Curves for AvrB alone and with 1 mM nucleotide of ADP, GDP, UDP, CDP, or dTDP. (C) Curves for AvrB alone and with RIN4 peptide Pcry (18 μM) in the absence or presence of 1 mM nucleotide of UDP, CDP, dTDP, or dNTPs (1 mM each). Calculated melting temperature for each curve is indicated.

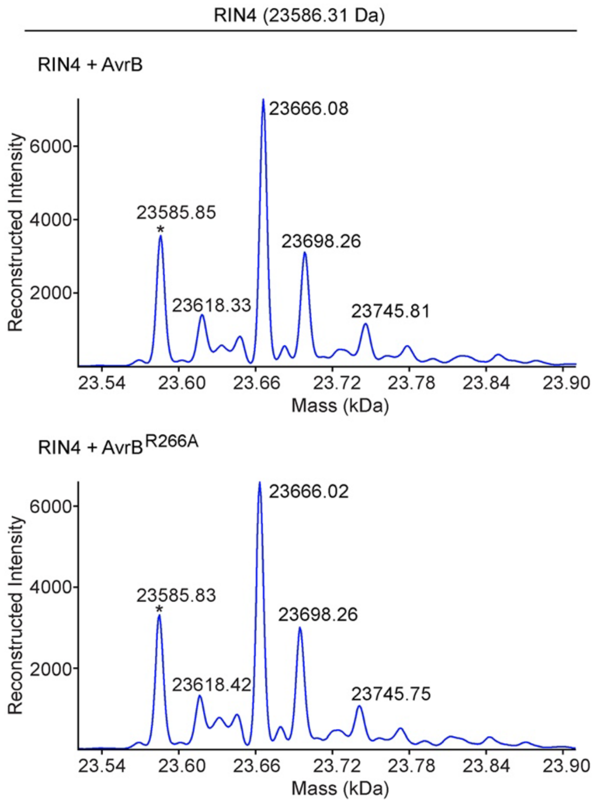

Figure S4. Intact mass analysis of RIN4 co-expressed with AvrB in human cells. RIN4 was co-expressed with AvrB or AvrB^{R266A} in HEK 293T/17 cells. "*" symbols indicate mass peaks close to the theoretical mass.

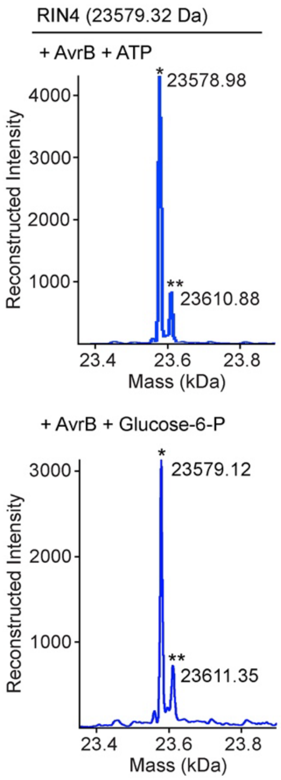

Figure S5. Intact mass of RIN4 from *in vitro* reaction assay. RIN4 was incubated with AvrB and co-substrate (ATP or Glucose-6-P), similarly as in Fig. 4D. "*" and "**" symbols indicate similar RIN4 peaks as in Fig. 1B.

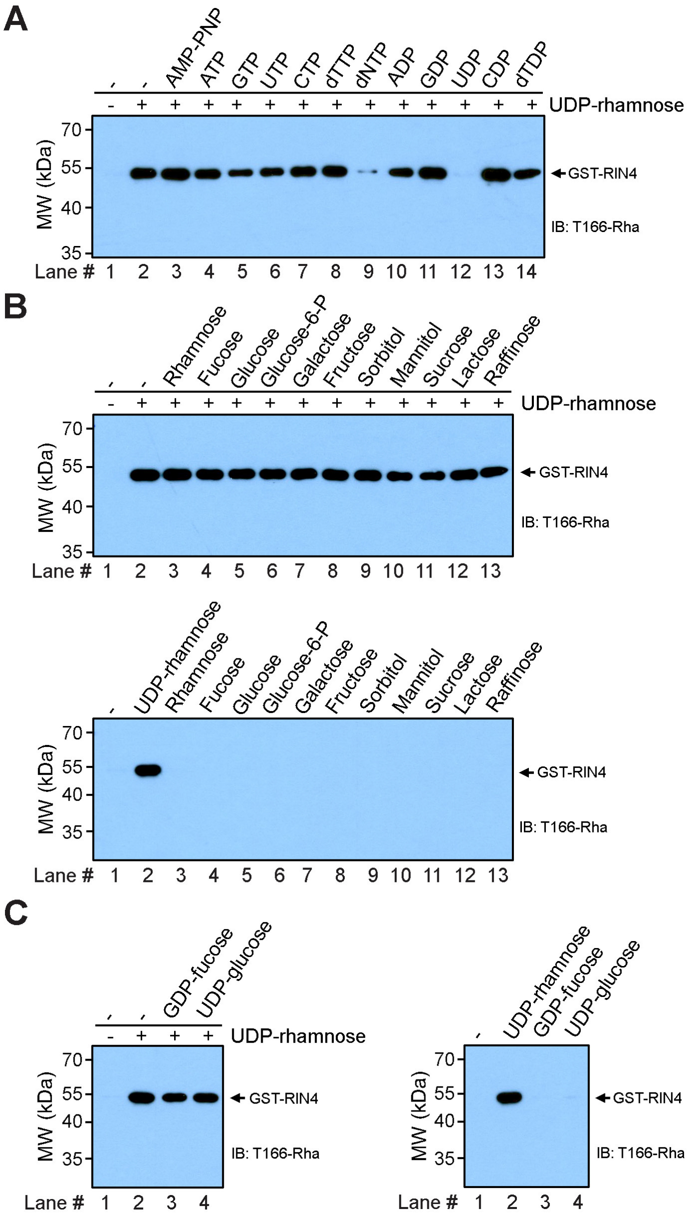

Figure S6. RIN4 rhamnosylation inhibition assay. (A) *In vitro* rhamnosylation assay in the presence of various nucleotides (1 mM) with 0.2 μM UDP-rhamnose. (B) *In vitro* rhamnosylation assay in the presence of various sugar or sugar-like molecules (1 mM) with (top) and without (bottom) 0.2 μM UDP-rhamnose. (C) *In vitro* rhamnosylation assay in the presence of 1 mM GDP-fucose or UDP-glucose with (left) and without (right) 0.2 μM UDP-rhamnose.

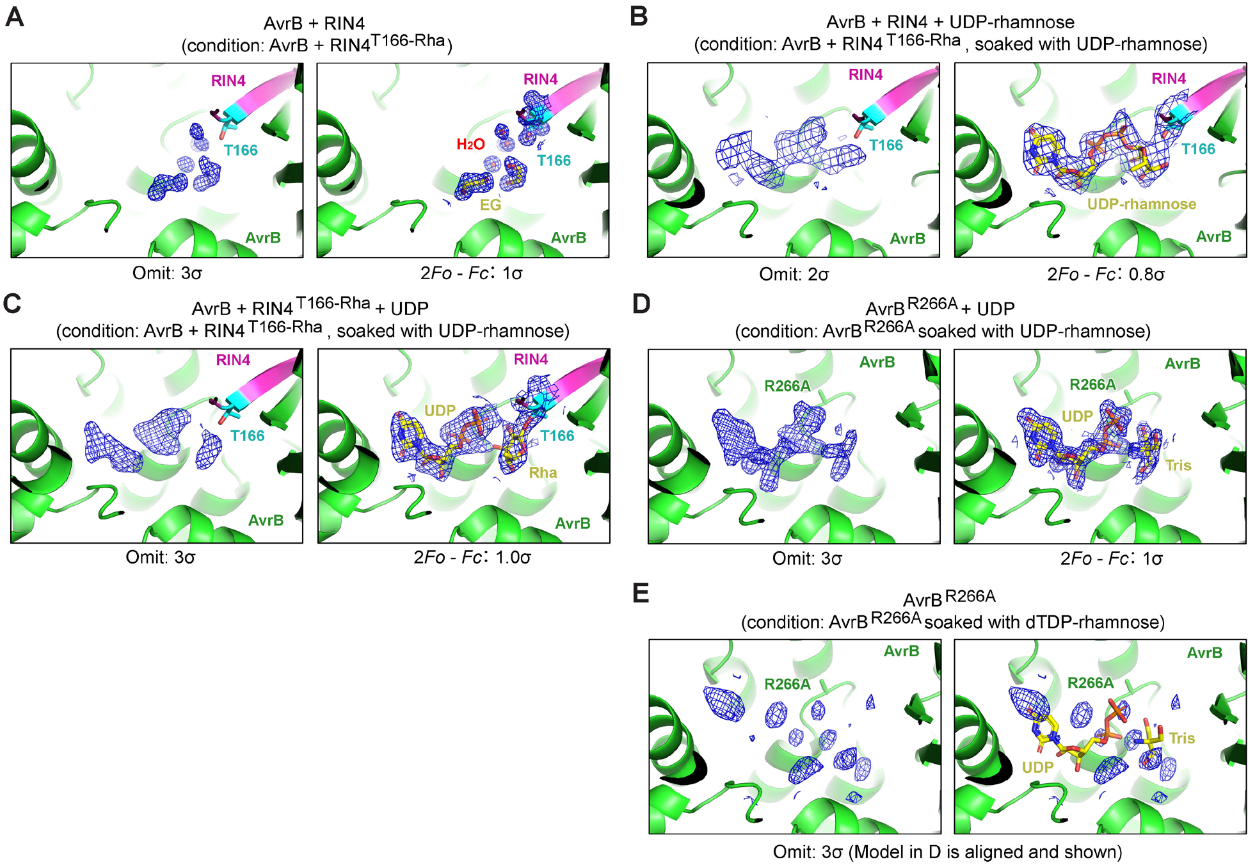

Figure S7. Electron densities of co-substrates bound in the pocket of AvrB. (A-E) The omit electron density map and the $2Fo - Fc$ electron density map for each co-substrate in the structure model, shown as blue mesh. Crystallization conditions are indicated for the structure models.

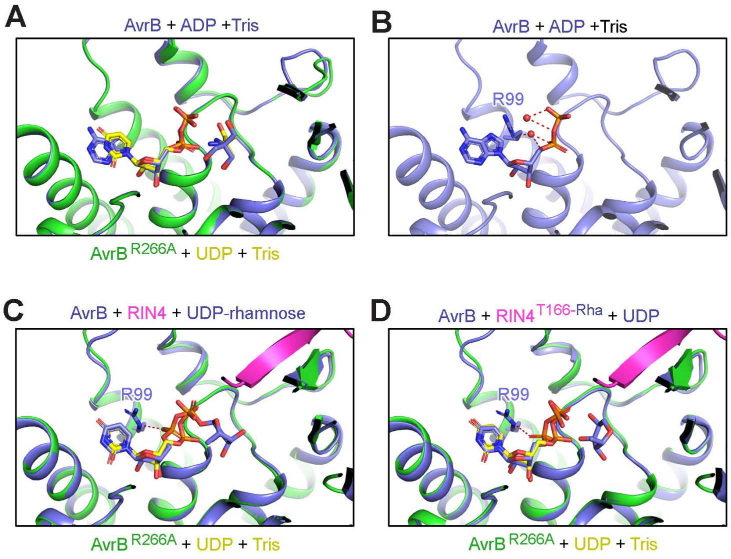

Figure S8. Conformations of nucleotide-containing molecules in structures of AvrB. (A-D) Comparison of nucleotide conformations in various AvrB structure models bound with co-substrates.

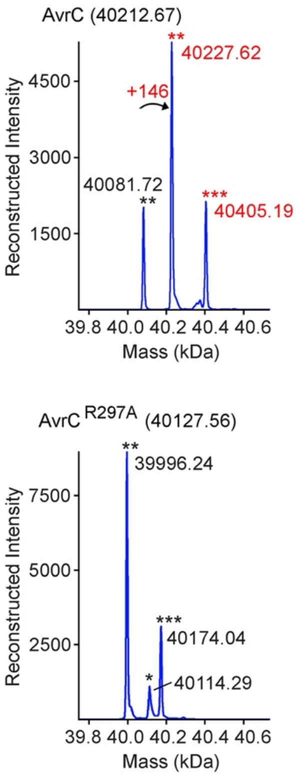

Figure S9. Intact mass analysis of AvrC. AvrC or AvrC^{R297A} (corresponding to AvrB^{R266A}) were expressed and purified from BL21 (DE3). "*" symbol in black indicates mass peak close to the theoretical mass; "**" symbols in black indicate peaks likely caused by removal of initiator methionine; "***" in black symbols indicate peaks likely caused by gluconoylation (compared to "**"). "**" and "***" in red indicate likely rhamnosylated protein peaks.

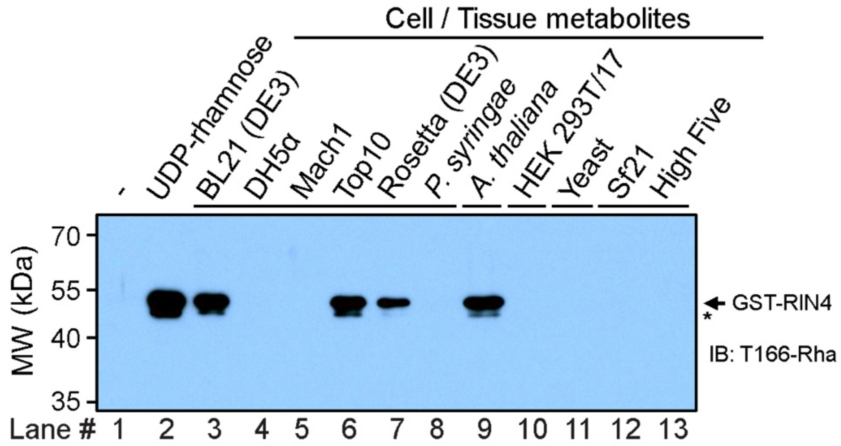

Figure S10. AvrB may rhamnosylate RIN4 *in planta*. RIN4 rhamnosylation by AvrB with cell/tissue metabolites as nucleotide sugar sources (or 0.2 μM UDP-rhamnose). Cell/tissue sources: *E. coli* strain BL21 (DE3) carrying an irrelevant plasmid, *E. coli* strain DH5α carrying an irrelevant plasmid, *E. coli* strain Mach1 carrying an irrelevant plasmid, *E. coli* strain TOP10, *E. coli* strain Rosetta (DE3), *Pseudomonas syringae* pv. *tomato* DC3000D28E carrying an irrelevant plasmid, *Arabidopsis thaliana* (Col-0) leaves (5~6 weeks old), mammalian cell HEK 293T/17, yeast strain *Saccharomyces cerevisiae* (BY4741, VPH1 with TAP tag), insect cell (Sf21 or High Five).

Table S1 | Data collection and refinement statistics.

**Data collection**				
Crystal	AvrB + RIN4 (8TXF)	AvrB + RIN4 + UDP-rhamnose (8TWS)	AvrB + RIN4$^{T166\text{-}Rha}$ + UDP (8TWO)	AvrB^{R266A} + UDP (8TWJ)
Wavelength (Å)	0.97918	1.0358	1.0358	0.97918
Resolution range (Å)	50.00 - 1.29 (1.31 - 1.29)	52.92 - 2.85 (2.90 - 2.85)	52.04 - 2.09 (2.13 - 2.09)	50.00 - 1.90 (1.93 - 1.90)
Space group	$P2_12_12$	$P2_12_12$	$P\ 2_12_12$	$P6_5$
Unit cell constants a, b, c (Å)	57.80, 119.90, 46.43	58.62, 123.58, 45.55	57.99, 119.24, 39.98	119.83, 119.83, 63.91
Total reflections	862,342	48,750 (2,215)	107,032 (5,420)	512,921
Unique reflections	82,030	8,193 (375)	16,986 (828)	41,240
Completeness (%)	99.9 (100.0)	99.9 (100.0)	99.9 (100.0)	99.7 (96.5)
Mean I/sigma(I)	46.1 (4.2)	2.0 (0.4)	6.6 (0.4)	26.4 (0.9)
Wilson B-factor (Å^2)	13.57	60.96	49.65	19.73
R-merge (%)	0.055 (0.596)	0.474 (2.001)	0.120 (2.307)	0.104 (1.326)
$CC_{1/2}$	(0.910)	0.925 (0.038)	0.997 (0.233)	(0.459)
**Refinement**				
Reflections for refinement	81,172 (7,654)	7,757 (663)	16,606 (1,467)	33,640 (1,180)
Reflections for R-free	1,991 (189)	777 (66)	1,661 (147)	2,007 (79)
Resolution range (Å)	34.65 - 1.29 (1.34 - 1.29)	52.97 - 2.85 (2.95 - 2.85)	52.15 - 2.09 (2.17 - 2.09)	40.28 - 1.90 (1.97 - 1.90)
Completeness (%)	98.95 (94.92)	94.86 (85.33)	97.7 (88.9)	81.44 (28.62)
R-work (%)	16.59 (19.20)	27.97 (38.05)	22.06 (40.86)	16.38 (20.82)
R-free (%)	17.99 (19.16)	33.54 (39.37)	28.97 (43.66)	19.13 (24.75)
Atoms (non-hydrogen)	2,938	2,550	2,550	2,705
macromolecules	2,544	2,515	2,540	2,426
ligands	150	57	21	40
solvent	334	0	0	263
R.m.s.d. bond (Å)	0.009	0.004	0.017	0.012
R.m.s.d. angle (°)	0.98	0.74	1.76	1.16
Ramachandran favored (%)	98.73	86.67	89.84	98.01
Ramachandran allowed (%)	0.95	12.06	9.21	1.66
Ramachandran outliers (%)	0.32	1.27	0.95	0.33
Rotamer outliers (%)	0.00	1.89	3.02	0.39
Clashscore	3.90	12.67	18.91	3.96
Average B-factor (Å^2)	22.59	66.69	76.67	27.18
macromolecules	21.50	66.78	76.80	26.19
ligands	27.94	60.59	69.76	38.96
solvent	29.91	-	-	35.44

Number of TLS groups	1	1	1	1

Statistics for the highest-resolution shell are shown in parentheses. $R_{merge}=\Sigma_h\Sigma_i|I_{h,i}-I_h|/\Sigma_h\Sigma_i I_{h,i}$, where I_h is the mean intensity of the i observations of symmetry related reflections of h. $R=\Sigma|F_{obs}-F_{calc}|/\Sigma F_{obs}$, where F_{calc} is the calculated protein structure factor from the atomic model).

REFERENCES AND NOTES

1. N. M. Alto, K. Orth, Subversion of cell signaling by pathogens. *Cold Spring Harb. Perspect. Biol.* **4**, a006114 (2012).

2. M. R. Popoff, D. Ladant, x*Comprehensive Sourcebook of Bacterial Protein Toxins, 4th Edition*, Xvii-Xviii (2015).

3. J. D. Jones, R. E. Vance, J. L. Dangl, Intracellular innate immune surveillance devices in plants and animals. *Science* **354**, (2016).

4. Y. Xiong, Z. Han, J. Chai, Resistosome and inflammasome: Platforms mediating innate immunity. *Curr. Opin. Plant Biol.* **56**, 47–55 (2020).

5. J. Shi, W. Gao, F. Shao, Pyroptosis: Gasdermin-mediated programmed necrotic cell death. *Trends Biochem. Sci.* **42**, 245–254 (2017).

6. J. D. Jones, J. L. Dangl, The plant immune system. *Nature* **444**, 323–329 (2006).

7. D. Mackey, B. F. Holt, 3rd, A. Wiig, J. L. Dangl, RIN4 interacts with Pseudomonas syringae type III effector molecules and is required for RPM1-mediated resistance in Arabidopsis. *Cell* **108**, 743–754 (2002).

8. M. J. Axtell, B. J. Staskawicz, Initiation of RPS2-specified disease resistance in Arabidopsis is coupled to the AvrRpt2-directed elimination of RIN4. *Cell* **112**, 369–377 (2003).

9. D. Mackey, Y. Belkhadir, J. M. Alonso, J. R. Ecker, J. L. Dangl, Arabidopsis RIN4 is a target of the type III virulence effector AvrRpt2 and modulates RPS2-mediated resistance. *Cell* **112**, 379–389 (2003).

10. T. J. Redditt, E. H. Chung, H. Z. Karimi, N. Rodibaugh, Y. Zhang, J. C. Trinidad, J. H. Kim, Q. Zhou, M. Shen, J. L. Dangl, D. Mackey, R. W. Innes, AvrRpm1

functions as an ADP-Ribosyl transferase to modify NOI Domain-containing proteins, including arabidopsis and soybean RPM1-interacting protein4. *Plant Cell* **31**, 2664–2681 (2019).

11. S. Choi, M. Prokchorchik, H. Lee, R. Gupta, Y. Lee, E. H. Chung, B. Cho, M. S. Kim, S. T. Kim, K. H. Sohn, Direct acetylation of a conserved threonine of RIN4 by the bacterial effector HopZ5 or AvrBsT activates RPM1-dependent immunity in Arabidopsis. *Mol. Plant* **14**, 1951–1960 (2021).

12. G. Zhao, D. Guo, L. Wang, H. Li, C. Wang, X. Guo, Functions of RPM1-interacting protein 4 in plant immunity. *Planta* **253**, 11 (2021).

13. B. Staskawicz, D. Dahlbeck, N. Keen, C. Napoli, Molecular characterization of cloned avirulence genes from race 0 and race 1 of Pseudomonas syringae pv. glycinea. *J. Bacteriol.* **169**, 5789–5794 (1987).

14. S. Gopalan, D. W. Bauer, J. R. Alfano, A. O. Loniello, S. Y. He, A. Collmer, Expression of the Pseudomonas syringae avirulence protein AvrB in plant cells alleviates its dependence on the hypersensitive response and pathogenicity (Hrp) secretion system in eliciting genotype-specific hypersensitive cell death. *Plant Cell* **8**, 1095–1105 (1996).

15. C. C. Lee, M. D. Wood, K. Ng, C. B. Andersen, Y. Liu, P. Luginbühl, G. Spraggon, F. Katagiri, Crystal structure of the type III effector AvrB from Pseudomonas syringae. *Structure* **12**, 487–494 (2004).

16. L. N. Kinch, M. L. Yarbrough, K. Orth, N. V. Grishin, Fido, a novel AMPylation domain common to fic, doc, and AvrB. *PLOS One* **4**, e5818 (2009).

17. E. H. Chung, L. da Cunha, A. J. Wu, Z. Gao, K. Cherkis, A. J. Afzal, D. Mackey, J. L. Dangl, Specific threonine phosphorylation of a host target by two unrelated type III effectors activates a host innate immune receptor in plants. *Cell Host Microbe* **9**, 125–136 (2011).

18. J. Liu, J. M. Elmore, Z. J. Lin, G. Coaker, A receptor-like cytoplasmic kinase phosphorylates the host target RIN4, leading to the activation of a plant innate immune receptor. *Cell Host Microbe* **9**, 137–146 (2011).

19. D. Desveaux, A. U. Singer, A. J. Wu, B. C. McNulty, L. Musselwhite, Z. Nimchuk, J. Sondek, J. L. Dangl, Type III effector activation via nucleotide binding, phosphorylation, and host target interaction. *PLOS Pathog.* **3**, e48 (2007).

20. H. Cui, Y. Wang, L. Xue, J. Chu, C. Yan, J. Fu, M. Chen, R. W. Innes, J. M. Zhou, Pseudomonas syringae effector protein AvrB perturbs Arabidopsis hormone signaling by activating MAP kinase 4. *Cell Host Microbe* **7**, 164–175 (2010).

21. N. Xu, X. Luo, W. Li, Z. Wang, J. Liu, The bacterial effector AvrB-induced RIN4 hyperphosphorylation is mediated by a receptor-like cytoplasmic kinase complex in arabidopsis. *Mol. Plant Microbe Interact.* **30**, 502–512 (2017).

22. A. R. Russell, T. Ashfield, R. W. Innes, Pseudomonas syringae effector AvrPphB Suppresses AvrB-induced activation of RPM1 but not AvrRpm1-induced activation. *Mol. Plant Microbe Interact.* **28**, 727–735 (2015).

23. J. W. Cruz, N. A. Woychik, Teaching fido new ModiFICation tricks. *PLOS Pathog.* **10**, e1004349 (2014).

24. C. R. Roy, J. Cherfils, Structure and function of Fic proteins. *Nat. Rev. Microbiol.* **13**, 631–640 (2015).

25. M. L. Yarbrough, Y. Li, L. N. Kinch, N. V. Grishin, H. L. Ball, K. Orth, AMPylation of Rho GTPases by Vibrio VopS disrupts effector binding and downstream signaling. *Science* **323**, 269–272 (2009).

26. H. Ham, A. R. Woolery, C. Tracy, D. Stenesen, H. Krämer, K. Orth, Unfolded protein response-regulated Drosophila Fic (dFic) protein reversibly AMPylates BiP chaperone during endoplasmic reticulum homeostasis. *J. Biol. Chem.* **289**, 36059–36069 (2014).

27. A. Sanyal, A. J. Chen, E. S. Nakayasu, C. S. Lazar, E. A. Zbornik, C. A. Worby, A. Koller, S. Mattoo, A novel link between Fic (filamentation induced by cAMP)-mediated adenylylation/AMPylation and the unfolded protein response. *J. Biol. Chem.* **290**, 8482–8499 (2015).

28. A. K. Casey, K. Orth, Enzymes Involved in AMPylation and deAMPylation. *Chem. Rev.* **118**, 1199–1215 (2018).

29. D. Castro-Roa, A. Garcia-Pino, S. de Gieter, N. A. J. van Nuland, R. Loris, N. Zenkin, The Fic protein Doc uses an inverted substrate to phosphorylate and inactivate EF-Tu. *Nat. Chem. Biol.* **9**, 811–817 (2013).

30. S. Mukherjee, X. Liu, K. Arasaki, J. McDonough, J. E. Galán, C. R. Roy, Modulation of Rab GTPase function by a protein phosphocholine transferase. *Nature* **477**, 103–106 (2011).

31. V. Campanacci, S. Mukherjee, C. R. Roy, J. Cherfils, Structure of the Legionella effector AnkX reveals the mechanism of phosphocholine transfer by the FIC domain. *EMBO J.* **32**, 1469–1477 (2013).

32. F. Feng, F. Yang, W. Rong, X. Wu, J. Zhang, S. Chen, C. He, J. M. Zhou, A Xanthomonas uridine 5′-monophosphate transferase inhibits plant immune kinases. *Nature* **485**, 114–118 (2012).

33. Y. Shang, X. Li, H. Cui, P. He, R. Thilmony, S. Chintamanani, J. Zwiesler-Vollick, S. Gopalan, X. Tang, J. M. Zhou, RAR1, a central player in plant immunity, is targeted by Pseudomonas syringae effector AvrB. *Proc. Natl. Acad. Sci. U.S.A.* **103**, 19200–19205 (2006).

34. L. E. Ong, R. W. Innes, AvrB mutants lose both virulence and avirulence activities on soybean and Arabidopsis. *Mol. Microbiol.* **60**, 951–962 (2006).

35. M. Varadi, S. Anyango, M. Deshpande, S. Nair, C. Natassia, G. Yordanova, D. Yuan, O. Stroe, G. Wood, A. Laydon, A. Žídek, T. Green, K. Tunyasuvunakool, S. Petersen, J. Jumper, E. Clancy, R. Green, A. Vora, M. Lutfi, M. Figurnov, A. Cowie, N. Hobbs, P. Kohli, G. Kleywegt, E. Birney, D. Hassabis, S. Velankar, AlphaFold protein structure database: Massively expanding the structural coverage of protein-sequence space with high-accuracy models. *Nucleic Acids Res.* **50**, D439-D444 (2022).

36. H. S. Kim, *D.* Desveaux, *A. U.* Singer, *P.* Patel, *J.* Sondek, *J. L.* Dangl, The pseudomonas syringae effector AvrRpt2 cleaves its C-terminally acylated target, RIN4, from Arabidopsis membranes to block RPM1 activation. *Proc. Natl. Acad. Sci. U.S.A.* **102**, 6496–6501 (2005).

37. B. A. Wagstaff, A. Zorzoli, H. C. Dorfmueller, NDP-rhamnose biosynthesis and rhamnosyltransferases: Building diverse glycoconjugates in nature. *Biochem. J.* **478**, 685–701 (2021).

38. N. Jiang, F. M. Dillon, A. Silva, L. Gomez-Cano, E. Grotewold, Rhamnose in plants - from biosynthesis to diverse functions. *Plant Sci.* **302**, 110687 (2021).

39. H. Kim, S. Kim, S. H. Yoon, Metabolic network reconstruction and phenome analysis of the industrial microbe, Escherichia coli BL21(DE3). *PLOS One* **13**, e0204375 (2018).

40. J. Lassak, E. C. Keilhauer, M. Fürst, K. Wuichet, J. Gödeke, A. L. Starosta, J. M. Chen, L. Søgaard-Andersen, J. Rohr, D. N. Wilson, S. Häussler, M. Mann, K. Jung, Arginine-rhamnosylation as new strategy to activate translation elongation factor P. *Nat. Chem. Biol.* **11**, 266–270 (2015).

41. R. Krafczyk, J. Macošek, P. K. A. Jagtap, D. Gast, S. Wunder, P. Mitra, A. K. Jha, J. Rohr, A. Hoffmann-Röder, K. Jung, J. Hennig, J. Lassak, Structural basis for EarP-mediated arginine glycosylation of translation elongation factor EF-P. *MBio* **8**, (2017).

42. T. Sengoku, T. Suzuki, N. Dohmae, C. Watanabe, T. Honma, Y. Hikida, Y. Yamaguchi, H. Takahashi, S. Yokoyama, T. Yanagisawa, Structural basis of protein arginine rhamnosylation by glycosyltransferase EarP. *Nat. Chem. Biol.* **14**, 368–374 (2018).

43. C. He, *N.* Liu*, F.* Li*, X.* Jia*, H.* Peng*, Y.* Liu*, Y.* Xiao, Complex structure of pseudomonas aeruginosa arginine rhamnosyltransferase EarP with Its acceptor elongation factor P. *J. Bacteriol.* **201**, (2019).

44. L. L. Lairson, B. Henrissat, G. J. Davies, S. G. Withers, Glycosyltransferases: Structures, functions, and mechanisms. *Annu. Rev. Biochem.* **77**, 521–555 (2008).

45. S. Tamaki, D. Dahlbeck, B. Staskawicz, N. T. Keen, Characterization and expression of two avirulence genes cloned from Pseudomonas syringae pv. glycinea. *J. Bacteriol.* **170**, 4846–4854 (1988).

46. Y. Song, B. R. Lee, S. Cho, Y. B. Cho, S. W. Kim, T. J. Kang, S. C. Kim, B. K. Cho, Determination of single nucleotide variants in Escherichia coli DH5α by using short-read sequencing. *FEMS Microbiol. Lett.* **362**, (2015).

47. S. Cunnac, S. Chakravarthy, B. H. Kvitko, A. B. Russell, G. B. Martin, A. Collmer, Genetic disassembly and combinatorial reassembly identify a minimal functional repertoire of type III effectors in Pseudomonas syringae. *Proc. Natl. Acad. Sci. U.S.A.* **108**, 2975–2980 (2011).

48. M. H. Black, A. Osinski, M. Gradowski, K. A. Servage, K. Pawłowski, D. R. Tomchick, V. S. Tagliabracci, Bacterial pseudokinase catalyzes protein polyglutamylation to inhibit the SidE-family ubiquitin ligases. *Science* **364**, 787–792 (2019).

49. A. Osinski, M. H. Black, K. Pawłowski, Z. Chen, Y. Li, V. S. Tagliabracci, Structural and mechanistic basis for protein glutamylation by the kinase fold. *Mol. Cell* **81**, 4527–4539.e8 (2021).

50. W. Minor, M. Cymborowski, Z. Otwinowski, M. Chruszcz, HKL-3000: The integration of data reduction and structure solution – from diffraction images to an initial model in minutes *Acta Crystallogr. D Biol. Crystallogr.* **62**, 859–866 (2006).

51. G. Winter, xia2: An expert system for macromolecular crystallography data reduction. *J. Appl. Cryst.* **43**, 186–190 (2010).

52. G. Winter, D. G. Waterman, J. M. Parkhurst, A. S. Brewster, R. J. Gildea, M. Gerstel, L. Fuentes-Montero, M. Vollmar, T. Michels-Clark, I. D. Young, N. K. Sauter, G. Evans, DIALS: Implementation and evaluation of a new integration package. *Acta Crystallogr. D. Struct. Biol.* **74**, 85–97 (2018).

53. P. D. Adams, P. V. Afonine, G. Bunkóczi, V. B. Chen, I. W. Davis, N. Echols, J. J. Headd, L. W. Hung, G. J. Kapral, R. W. Grosse-Kunstleve, A. J. McCoy, N. W. Moriarty, R. Oeffner, R. J. Read, D. C. Richardson, J. S. Richardson, T. C. Terwilliger, P. H. Zwart, PHENIX: A comprehensive Python-based system for macromolecular structure solution. *Acta Crystallogr. D Biol. Crystallogr.* **66**, 213–221 (2010).

54. P. Emsley, B. Lohkamp, W. G. Scott, K. Cowtan, Features and development of Coot. *Acta Crystallogr. D Biol. Crystallogr.* **66**, 486–501 (2010).

55. V. B. Chen, W. B. Arendall III, J. J. Headd, D. A. Keedy, R. M. Immormino, G. J. Kapral, L. W. Murray, J. S. Richardson, D. C. Richardson, MolProbity: All-atom structure validation for macromolecular crystallography. *Acta Crystallogr. D Biol. Crystallogr.* **66**, 12–21 (2010).

56. L. Patiny, A. Borel, ChemCalc: A building block for tomorrow's chemical infrastructure. *J. Chem. Inf. Model.* **53**, 1223–1228 (2013).
